# Purinergic receptor P2Y12 boosts autoimmune hepatitis through hexokinase 2-dependent glycolysis in T cells

**DOI:** 10.7150/ijbs.85133

**Published:** 2023-07-09

**Authors:** Wei Zhuang, Xiucheng Liu, Guangyu Liu, Jie Lv, Hao Qin, Chun Wang, Ling Xie, Kaidireya Saimaier, Sanxing Han, Changjie Shi, Qiuhong Hua, Ru Zhang, Changsheng Du

**Affiliations:** 1 Key Laboratory of Spine and Spinal Cord Injury Repair and Regeneration of Ministry of Education, Orthopaedic Department of Tongji Hospital, School of Life Sciences and Technology, Tongji University, Shanghai 200092, China.; 2 Institute of Biophysics, Chinese Academy of Sciences, Beijing 100101, China.; 3 College of Life Sciences, University of Chinese Academy of Sciences, Beijing, 100049, China.; 4 Department of Thoracic Surgery, Shanghai Pulmonary Hospital, Tongji University School of Medicine, Shanghai, 200433, China.; 5 Department of Thoracic Surgery, Huadong Hospital Affiliated to FuDan University, Shanghai, 200040, China.

**Keywords:** P2RY12, autoimmune hepatitis, T cell metabolism, glycolysis, HK2

## Abstract

Increasing evidence suggests that immunometabolism has started to unveil the role of metabolism in shaping immune function and autoimmune diseases. In this study, our data show that purinergic receptor P2Y12 (P2RY12) is highly expressed in concanavalin A (ConA)-induced immune hepatitis mouse model and serves as a potential metabolic regulator in promoting metabolic reprogramming from oxidative phosphorylation to glycolysis in T cells. P2RY12 deficiency or inhibition of P2RY12 with P2RY12 inhibitors (clopidogrel and ticagrelor) are proved to reduce the expression of inflammatory mediators, cause CD4^+^ and CD8^+^ effector T cells hypofunction and protect the ConA-induced immune hepatitis. A combined proteomics and metabolomics analysis revealed that P2RY12 deficiency causes redox imbalance and leads to reduced aerobic glycolysis by downregulating the expression of hexokinase 2 (HK2), a rate-limiting enzyme of the glycolytic pathway, indicating that HK2 might be a promising candidate for the treatment of diseases associated with T cell activation. Further analysis showed that P2RY12 prevents HK2 degradation by activating the PI3K/Akt pathway and inhibiting lysosomal degradation. Our findings highlight the importance of the function of P2RY12 for HK2 stability and metabolism in the regulation of T cell activation and suggest that P2RY12 might be a pivotal regulator of T cell metabolism in ConA-induced immune hepatitis.

## Introduction

The liver is a major and frontline immunological organ containing a large number of innate and adaptive immune cells that play a role in coordinating immune responses^1^, ^2^. Immune-mediated hepatitis occurs when the liver is attacked by the body's immune system causing inflammation and ultimately cirrhosis, as well as when virus-specific T cells destroy infected hepatocytes in viral hepatitis^3^. In autoimmune hepatitis (AIH), T cells control the progression of the disease by interacting with other immune cells including B cells, dendritic cells (DCs), macrophages, and natural killer (NK) cells^4^, ^5^. Intravenous injection of concanavalin A (ConA) into mice has been widely used to establish a mouse model of fulminant immunological liver injury* in vivo*^6^-^8^. In this model, intravenous administration of ConA rapidly activates T cells, such as CD4^+^ T cells, CD8^+^ T cells, natural killer T (NKT) cells and NK cells, which leads to the production of a variety of proinflammatory cytokines (e.g. tumor necrosis factor (TNF), IFN-γ, IL-6) that ultimately cause acute liver injury^9^, ^10^. The underlying mechanisms that promote progression of T cells are not yet completely understood, but accumulating evidence suggests that increased metabolism contributes to T cell activation in autoimmune diseases^11^-^13^.

In recent years there has been growing interest in understanding T cell metabolism due to its extremely dynamic nature^14^. Naive T cells are characterized by decreased metabolic activity, which is mainly catabolic, and primarily rely on oxidative phosphorylation (OXPHOS) and fatty acid oxidation (FAO)^15^. Once activated, T cells require large amounts of glucose, amino acids, and fatty acids to support cellular growth and clonal expansion^16^. To meet these demands, upon activation, T cells shift their metabolism from OXPHOS to primary glycolysis and Warburg metabolism for their energy supply^17^, ^18^. Besides supporting the faster ATP generation, glycolysis and Warburg metabolism provide more critical intermediary metabolites to promote T cell differentiation^19^. Of note, the activity of metabolic enzymes or nutrient transporters influences the differentiation and function of T cells in autoimmune responses. For instance, inhibiting GLUT3-dependent acetyl-CoA production alleviates Th17-cell-mediated inflammatory diseases^12^, while targeting pyruvate kinase activity restrains excessive activation of CD4^+^ T cells and prevents the development of autoimmunity 20. In rheumatoid arthritis (RA) patients, the metabolic signature of T cells is characterized by the shunting of glucose toward the pentose phosphate pathway and toward biosynthetic activity^21^. Moreover, high energy demands and a switch toward cellular glycolysis from OXPHOS for energy is the metabolic features of systemic lupus erythematosus (SLE) T cells^22^, ^23^. In ConA-induced immune hepatitis, glucose metabolism reprogramming promotes AIH development by reducing and impairing the function regulatory T cells (Treg) population^24^. Although this represents an important advance in explaining the progression of AIH, the molecular mechanisms linking T cell signaling and metabolic activities are still largely unknown.

The purinergic receptor P2Y12 (P2RY12) is an ADP-responsive G protein-coupled receptor that is widely expressed in many cells, including on the surface of platelets^25^, ^26^. Binding of ADP to Gi-coupled P2RY12 activates multiple biochemical pathways to amplify platelet activation, aggregation and thrombus formation through pathways involving activation of PI3 kinase (PI3K) and its downstream kinase Akt^27^. In addition to its well-known antiplatelet effect, P2RY12 also has anti-atherosclerotic and neuroprotective effects by targeting other cells, such as endothelial cells, DCs, and T lymphocytes^28^-^30^. In DCs, it increases macro-pinocytosis in murine DCs, which leads to their enhanced ability to stimulate Ag-specific T cells^29^. P2RY12 activation has effect on biological responses of T cells to stimulation in a time-dependent and stimulus-type-specific manner^31^. We have previously shown that the expression of P2RY12 was markedly upregulated in peripheral lymphoid tissues of experimental allergic encephalomyelitis (EAE) mice, and its deletion suppressed Th17 cell differentiation and attenuated autoimmune disease progression^32^. However, how and to what extent P2RY12 influences T lymphocytes and contributes to further immune-mediated injury has not yet been fully elucidated.

In this study, we hypothesized that P2RY12 is of unique significance to T lymphocytes in AIH disease progression. To confirm the validity of this hypothesis, we systematically evaluated the effects of P2RY12 on T lymphocytes in ConA-induced immune hepatitis* in vivo* and* in vitro*. A combined proteomics and metabolomic analysis were performed to determine the role of P2RY12 and the detailed underlying mechanism. Additionally, we found that P2RY12 functions post-translationally by mediating the stabilization of hexokinase 2 (HK2), a rate-limiting enzyme of the glycolytic pathway. P2RY12 prevents the degradation of HK2 by controlling lysosomal degradation and activating Akt phosphorylation. These findings suggest that P2RY12 function as a pivotal regulator of T cell metabolism and identify a novel link between glycolysis and AIH pathogenesis.

## Methods and Materials

### Mice

C57BL/6 mice were purchased from GemPharmatech (Nanjing, China). P2RY12^-/-^ mice on a C57BL/6 background were described previously^33^. Rag1^-/-^ mice on C57BL/6 background were obtained from The Jackson Laboratory (Bar Harbor, ME, USA). All mice were housed under specific pathogen-free conditions and fed with standard laboratory chow and water ad libitum. All animal experiments were performed in accordance with the guidelines of Tongji University Animal Care Committee and the Animal Research Ethics Committee.

### ConA-induced immune hepatitis

A single dose of ConA (Sigma-Aldrich, St. Louis, MO) was dissolved in normal saline solution at a concentration of 10 mg/ml. At the age of 8-10 weeks, mice were intravenously injected with ConA at 7.5, 10, 12.5, 15 or 20 mg/kg body weight, through the tail vein and their survival rate was monitored continuously for 7 days. The ConA dose of 12.5 mg/kg body weight injected intravenously was selected to induce immune hepatitis in mice. Clopidogrel (Sanofi Clir SNC, France) (7.5, 15, 30 mg/kg body weight) or ticagrelor (AstraZeneca AB, Sweden) (15, 30, 60 mg/kg body weight) was administered orally once daily for 7 days before ConA treatment. Clopidogrel (15 mg/kg body weight) or ticagrelor (15 mg/kg body weight) was administered orally once after ConA treatment 30 min, 1 hr or 12 hr. A 0.9% saline solution was used as vehicle.

### Biochemical and Histological Analyses

Blood was collected from the post-orbital venous plexus vein at different times after the ConA injection, and the serum was separated by centrifugation at 4,000×g at 4 °C for 10 min. Serum levels of alanine aminotransferase test (ALT) and aspartate aminotransferase test (AST) were measured using reagent kits (ADICON, Shanghai, China) following the manufacturer's specifications. For histological staining, mice were anesthetized and perfused with phosphate-buffered saline (PBS), followed by fixation with 4% (w/v) paraformaldehyde. The isolated livers tissues were then fixed in 4% (w/v) paraformaldehyde for 24 hr, embedded in paraffin, sectioned into 4-µm-thick sections, stained with hematoxylin and eosin (H&E), and examined for the presence of inflammation by light microscopy using an inverted Olympus IX51 microscope (Olympus Corporation, Tokyo, Japan).

### Human Peripheral Blood Mononuclear Cell Isolation

The samples of human blood were obtained from healthy volunteers and AIH patients following the procedures and guidelines approved by the Institutional Review Board of Tongji University School of Medicine. All procedures followed were in accordance with the ethical standards of the responsible committee on human experimentation (the institutional review board at Tongji University) and with the Helsinki Declaration of 1975, as revised in 2000. Informed consent was obtained from all subjects for being included in the study.

Peripheral blood mononuclear cells (PBMCs) were isolated as previously described^34^. Briefly, total blood was diluted with PBS (1:1), and 6 ml of diluted blood was gently mixed with 3 ml of Lymphoprep^TM^ density gradient solution (Serumwerk Bernburg AG, Oslo, Norway), and centrifuged at 800×g for 30 min at room temperature. Cells were collected from the interphase layer, and the collected fraction was diluted with PBS and centrifuged at 1,000×g for 5 min. Collected cells were transferred to RPMI 1640 medium supplemented with 10% fetal bovine serum (FBS), penicillin-streptomycin (100 ×) and glutamine (2 mM) and maintained at 37 °C in a humidified atmosphere containing 5% CO_2_. The obtained PBMCs were stimulated with 2 μg/ml of ConA in the culture medium except for the negative control group, other groups were treated with different appropriate treatments. After 24 hr, cells and supernatants were collected for flow cytometry, RT-qPCR and enzyme-linked immunosorbent assay (ELISA) analysis.

### Isolation of Hepatic lymphocytes

Hepatic lymphocytes were prepared as previously described^3^, ^35^. Briefly, each liver was removed and pressed through 100-µm mesh cell strainer (BD Falcon, Bedford, MA, USA). The collected cells were centrifuged at 500×g for 3 min, then the cell pellet was resuspended in 40% Percoll and then overlaid on 70% Percoll and centrifuged at 2400 rpm for 25 min at room temperature. Hepatic lymphocytes were collected from the interphase. Eventually, the resulting pellet was collected and resuspended in 1.5 ml of red blood cell lysing buffer for 5 min, centrifuged at 500×g for 3 min, washed twice in PBS and then the hepatic lymphocytes were counted.

### RNA Isolation and Quantitative Real-Time Polymerase Chain Reaction

12 hr after injection with ConA, mice were euthanized and liver tissues were harvested, and used for total RNA extraction with TRIzol reagent (Invitrogen, Carlsbad, CA, USA). After synthesizing cDNA from the RNA samples, cDNA samples were subjected to quantitative real-time polymerase chain reaction (qRT-PCR) using the TaqMan^TM^ Universal PCR Master Mix (Applied Biosystems, Foster City, CA, USA) and TaqMan^TM^ Gene Expression Assays (Applied Biosystems) following the manufacturers' instructions. QRT-PCR analysis was conducted in a Light Cycler quantitative PCR apparatus (Stratagene, Santa Clara, CA, USA) using the SYBR Green JumpStart Taq ReadyMix kit (Sigma-Aldrich). The expression value was normalized to β-actin in the same sample. The primers used in the PCR reactions, including those for mouse *Il6*, *Il12a, Il10*, *Tnf*, *Ifng*, and* Actb* and for human *IL-6*, *IL-12A*,* IL10*, *TNF*, *IFNG* and *ACTB* (Supplementary Table 1) were synthesized by Sangon Biotech (Shanghai, China).

### *In Vitro* Experiments

Splenocytes from wild type (WT) mice and P2RY12^-/-^ mice were isolated and cultured in 96-well plates at a density of 1×10^6^ cells per well, then stimulated with ConA (2 μg/ml) (Sigma-Aldrich) for 12 hr or 24 hr and co-cultured with the P2RY12 inhibitors clopidogrel (1, 3, 10 μM) or ticagrelor (1, 3, 10 μM), HK2 inhibitor 3-bromopyruvate (3-BrPA, MedKoo Biosciences Inc., Morrisville, NC, USA), proteasome inhibitor MG-132 (Med-Chem-Express LLC, Monmouth Junction, NJ, USA), lysosomal inhibitor bafilomycin A1 (BafA-1) (Med Chem Express LLC) or Akt activator SC79 (Med-Chem-Express LLC). The cells were analyzed by flow cytometry and supernatants were collected for measurement of cytokines.

### Flow Cytometry

Freshly isolated hepatic lymphocytes separated from liver tissue sections or human PBMCs were stained with various antibodies in the dark at 4°C for 30 min, then washed twice with PBS. After fixation and permeabilization with a Cytofix/Cytoperm kit (BD Biosciences, San Jose, CA, USA) and staining with various antibodies, including anti-mouse CD3-PE (100206, BioLegend), anti-mouse NK1.1-FITC (108706, BioLegend), anti-mouse CD8a-FITC (2002714, Invitrogen), anti-mouse CD4-PE-Cy7 (25-0042-82, eBioscience), anti-mouse IFN-γ-APC (505810, BioLegend), anti-human CD4-PE-Cy7 (25-0049-42, eBioscience), anti-human CD8a-FITC (2518338, Invitrogen), anti-human IFN-γ-APC (17-7319-82, eBioscience). The cells were analyzed by flow cytometry on a BD FACSVerse^TM^ Flow Cytometer (BD Bioscience) and the analysis was used by The Guava Soft software.

### Western blot analysis

To evaluate the expression levels of different proteins in liver, mouse livers were homogenized in radioimmunoprecipitation assay (RIPA) buffer containing a protease inhibitor cocktail (Roche Applied Science, Penzberg, Germany) and centrifuged at 10,000×g at 4 °C for 15 min. The proteins in the supernatants were solubilized by boiling for 5 min in sodium dodecyl sulfate-polyacrylamide gel electrophoresis (SDS-PAGE) sample-loading buffer. Then, 50 μg of solubilized proteins were separated on SDS-PAGE gels and subsequently electro-transferred onto a nitrocellulose membrane (Millipore Sigma, Burlington, MA, USA). After blocking the membrane with 5% nonfat milk to prevent nonspecific binding, immunoblotting was performed using the following antibodies against mouse proteins: GPI, PFK2, MCT1, TPI(Proteintech Inc., Rosemont, USA); JNK, p-JNK (Thr183/Tyr185), cleaved caspase3 and β-actin (Cell Signaling Technology, Danvers, MA, USA); P2RY12 (Abcam Inc., Cambridge, UK); HK2, P62 and LC3 (ABclonal, Wuhan, China); Akt, P-Akt (Ser473) (Proteintech Inc., Rosemont, USA); P-Akt (Thr308) (Affinity Biosciences, Cincinnati, OH, USA).

### Co-immunoprecipitation

Whole proteins of liver tissue were extracted and measured protein concentrations using the bicinchoninic acid (BCA) protein assay. Part of the supernatant was used as input control and the rest was immunoprecipitated overnight at 4ºC by gently rocking with anti-HK2 antibody (Abcam Inc., Cambridge, UK).

Approximately 5 μl antibodies were used for 500 μg total protein. Then protein A/G agarose beads (Santa Cruz Biotechnology Inc., Santa Cruz, CA, USA) were added to bind with the immunoprecipitates for 2 hr with gentle shaking at room temperature. Precipitated proteins were washed 3 times with PBS and boiled with 5×loading buffer, then immunoblotting was performed as previously described. Rabbit normal IgG (Santa Cruz Biotechnology Inc.) served as negative control.

### Measurement of cytokine levels

Individual mouse serum and culture supernatants were collected, and the levels of inflammatory cytokines were measured using an ELISA kit (BioLegend, San Diego, CA, USA). according to the manufacturer's instructions.

### Liquid chromatography tandem Mass spectrometry (LC-MS/MS)

Hepatic lymphocytes were isolated from the liver of WT mice and P2RY12^-/-^ mice with ConA-induced immune hepatitis and T cells were sorted by the Magnisort^TM^ mouse CD3 positive selection kit (Invitrogen, Carlsbad, CA, USA). The sorted T cells were lysed in 1,000 µl RIPA buffer at 4 °C. The BCA protein assay was used to determine the total protein content of each sample. Samples were analyzed on Orbitrap Fusion Lumos, and Q Exactive Plus mass spectrometers (Thermo Fisher Scientific, Rockford, IL) coupled with an EASY-nLC 1000 nanoflow liquid chromatograph system (Thermo Fisher Scientific) connected to an UltiMate 3000 RSLCnano system (Thermo Fisher Scientific).

### Metabolomics

Hepatic lymphocytes isolated from ConA-induced immune hepatitis and sorted T cells. T cells were washed twice with ammonium carbonate (75 mM) at pH 7.4 and snap frozen in liquid nitrogen. Metabolomics analysis was performed using a 1260 infinity high-performance liquid chromatography (HPLC) system (Agilent Technologies Inc., Santa Clara, CA, USA). Before LC/MS analysis, samples were prepared by different procedures and stored at -80°C^36^.

For LC/MS, samples were separated on an amide column, using water mixed with 25 mM ammonium acetate and 25 mM Ammonium hydroxide as mobile phase A and ACN as mobile phase B. MS analysis was performed on a UHPLC-Q-Exactive MS/MS system (Thermo Fisher Scientific Inc.) in both positive and negative ion modes. Different data-dependent analysis (DDA) methods and the full scan method were performed, and the results were processed using untargeted metabolomics workflow with minor modification to find and identify the differences between samples. The exact mass of each feature was submitted to selected ChemSpider with 4 databases (BioCyc, Human Metabolome Database, KEGG; Lipid MAPS) and then analyzed with *R* statistical software (http://www.r-project.org).

### Metabolic Assays

The oxygen consumption rate (OCR) and extracellular acidification rate (ECAR) were determined using a Seahorse XF96 Extracellular Flux Analyzer (Agilent Technologies Inc.) following established protocols^18^. Briefly, P2RY12^-/-^ mice and age- and sex-matched WT control mice (8-10 weeks) were used to establish the ConA-induced immune hepatitis model by injecting ConA into tail vein at the dose of 12.5 mg/ kg of body weight for 12 hr. Hepatic lymphocytes isolated and further sorted T cells with Magnisort^TM^ mouse CD3 positive selection kit (Invitrogen, Carlsbad, CA, USA). OCR was evaluated under basal conditions and in response to 1 μM oligomycin, 0.6 μM FCCP, 100 nM rotenone, plus 1 μM antimycin A. ECAR was measured under basal conditions and in response to 100 mM glucose, 1 μM oligomycin, and 5 mM 2-d-glucose (2-DG). Activated T cells were cultured in XF-medium (XF Base Medium Minimal Dulbecco's modified Eagle's medium (DMEM); Agilent Technologies Inc.) containing 10 mM glucose, 2 mM L-glutamine, and 1 mM sodium pyruvate. Assays were analyzed with the Wave Desktop software (Agilent Technologies Inc.).

### Measurement of ATP level

ATP production level was quantified by a luminescent available assay kit (Beyotime Biotechnology, Shanghai, China). Briefly, T cells isolated from liver tissues were lysed to release ATP into medium containing luciferase and luciferin, which produces light in the presence of ATP. The light emission, which is proportional to the amount of ATP present was detected.

### Statistical Analysis

Data are expressed as the means ± SEM and were analyzed using the Graphpad Prism software (version 7.0; GraphPad software Inc., CA, USA). Differences between groups were assessed by means of a two-tailed unpaired Student *t* test. For three or more groups, a one-way analysis of variance (ANOVA) was used, followed by Tukey *post-hoc* test. All the experiments were repeated at least three times. *P* < 0.05 was considered to be statistically difference.

## Results

### Deletion of P2RY12 protects mice from ConA-induced immune hepatitis

In this study, we first established a ConA-induced immune hepatitis mouse model with ConA intravenous injection. Consistent with previous reports, we found that ConA injection led to liver injury, increased serum alanine aminotransferase (ALT) and aspartate aminotransferase (AST) levels, and increased rate of death in a dose-dependent manner (Figure S1)^37^. A significant increase in the expression of P2RY12 was found at both protein and mRNA levels in mice liver with 12.5 mg/kg ConA intravenous injection (Figure S2A, S2B). Further investigation showed that the mRNA level of *P2ry12* was up-regulated in hepatic lymphocytes and down-regulated in hepatic cells, suggesting that hepatic lymphocytes are the main contributor to the increased P2RY12 level in the liver of mice with ConA injection (Figure S2C-E).

**Figure 1:**
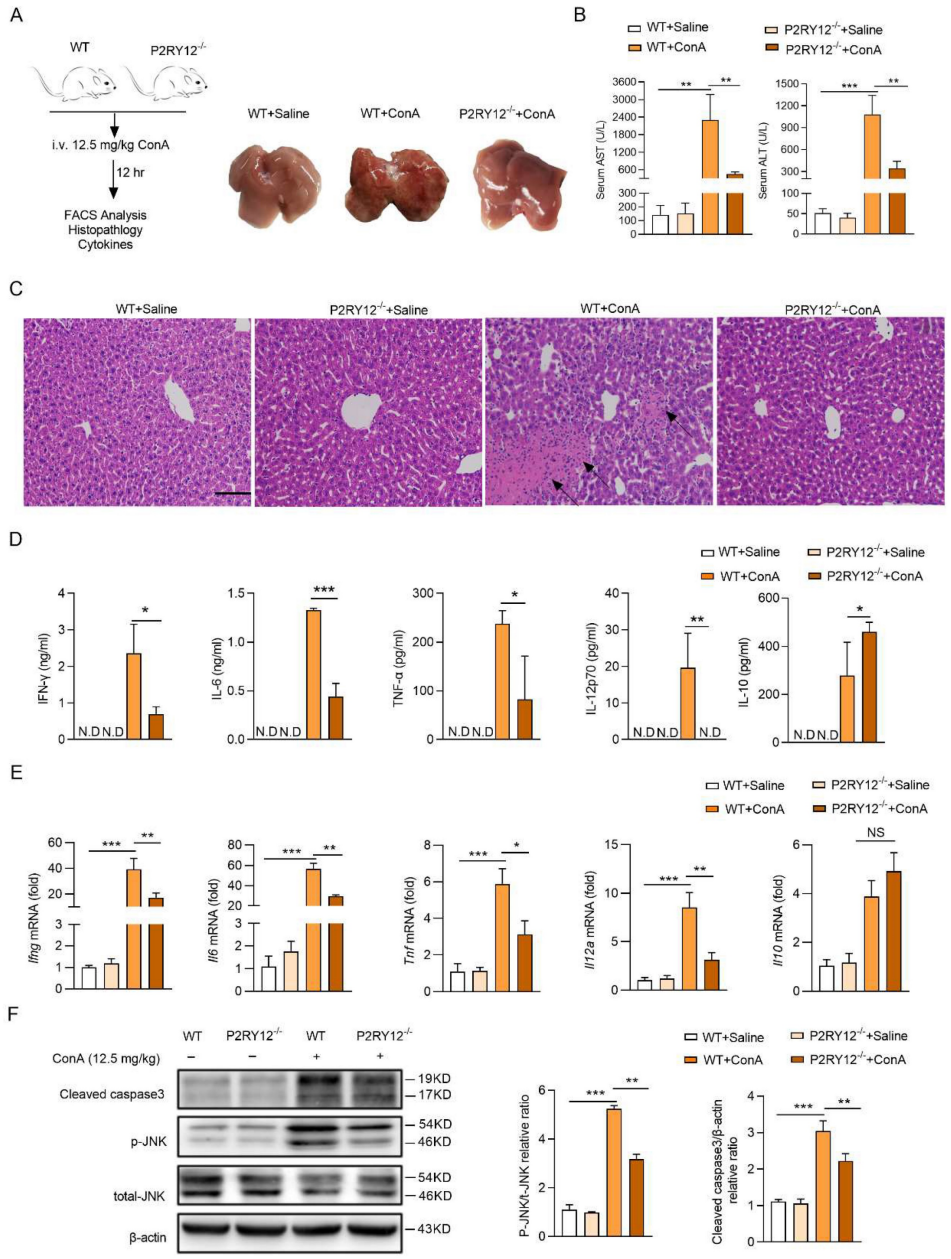
** P2RY12 deficiency protected from ConA-induced immune hepatitis.** (A) Schematic diagram of ConA-induced immune hepatitis mouse model. WT and P2RY12^-/-^ mice were administered ConA (12.5 mg/kg body weight via* i.v.* injection) for 12 hr (left). The livers were harvested and pictured from WT after saline for 12 hr, WT and P2RY12^-/-^ mice after 12.5 mg/kg ConA injection for 12 hr (right). (B) Serum levels of AST and ALT in WT mice and P2RY12^-/-^ mice were detected. (C) Photomicrographs of representative H&E-stained mouse livers. Massive hepatocyte necrosis (dark arrows) was observed in WT+ConA group. (D) ELISA analysis of cytokines (IFN-γ, IL-6, IL-12p70, TNF-α and IL-10) in the serum from WT and P2RY12^-/-^ mice without or with 12.5 mg/kg ConA injection for 12 hr. (E) mRNA levels of* Ifng*,* Il6*,* Il12a*,* Tnf* and *Il10* in liver tissue from WT and P2RY12^-/-^ mice were assessed. (F) Liver cleaved caspase-3, JNK and p-JNK protein levels were determined by Western blot (left). Pooled data are presented in the right panel. Data represented as the mean±SD of 4 mice per group. Data are mean ± SEM. *P < 0.05; **P < 0.01; ***P < 0.001* vs* indicated group. (two-tailed Student's t-test).

Next, we generated P2RY12 knockout (P2RY12^-/-^) mice to determine the role of P2RY12 in ConA-induced immune hepatitis (Figure 1A). Compared with WT mice, P2RY12^-/-^ mice showed relieved ConA-induced immune hepatitis and decreased serum ALT and AST levels (Figure 1B, 1C). Moreover, ConA injection led to significant increases of immune liver injury related serum cytokines, including IFN-γ, IL-12p70, IL-6, and TNF-α^38^. While P2RY12 knockout was able to partly block the ConA-induced increases of serum IFN-γ, IL-12p70, IL-6, and TNF-α, and decrease of serum IL-10 (Figure 1D). Consist with serum cytokine levels, the mRNA levels of cytokines in liver tissue showed similar tendency (Figure 1E). Apoptosis and phosphorylation of JNK are key factors during the pathological process of liver injury 39, 40. Accordingly, ConA administration markedly increased cleaved caspase-3 and phosphorylated of JNK in the liver of WT mice but not P2RY12^-/-^ mice (Figure 1F). We initially confirm that P2RY12 deletion protects mice from ConA-induced immune hepatitis.

### P2RY12 positively regulates the activation and activity of T cells

**Figure 2:**
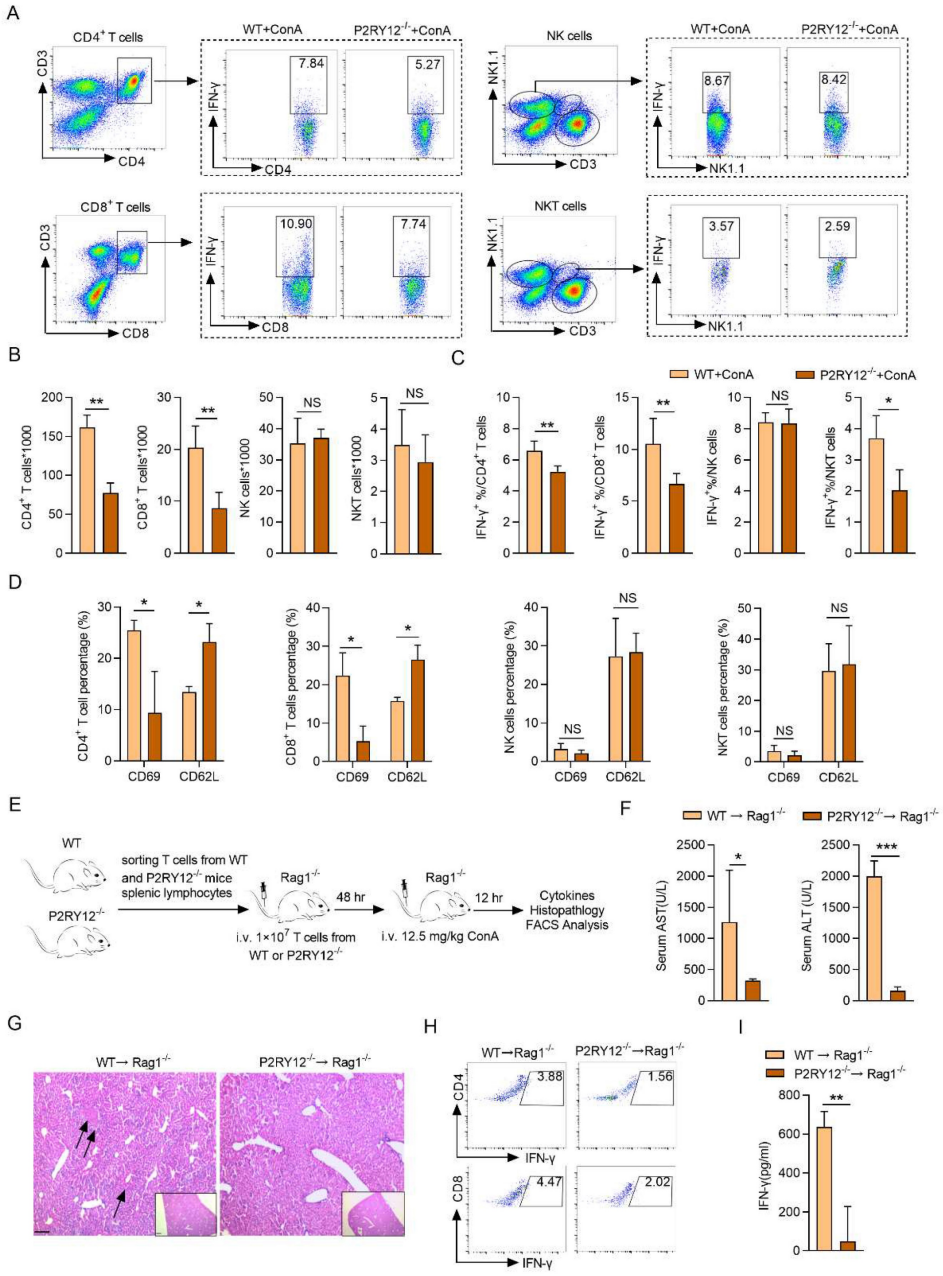
** P2RY12 in T cells exacerbates the pathology of ConA-induced immune hepatitis.** WT mice and P2RY12^-/-^ mice were administered ConA (12.5 mg/kg body weight via *i.v.* injection) for 12 hr. (A) IFN-γ expression in CD4^+^ T cells, CD8^+^ T cells, NK cells, and NKT cells from liver lymphocytes of WT mice and P2RY12^-/-^ mice were analyzed by flow cytometry. (B) Total numbers of liver lymphocytes, including CD4^+^ T cells, CD8^+^ T cells, NK cells, NKT cells were observed in the liver of WT mice and P2RY12^-/-^ mice. (C) The proportion of IFN-γ^+^ in CD4^+^ T cells, CD8^+^ T cells, NK cells, NKT cells of WT + ConA mice and P2RY12^-/-^ + ConA mice were examined by flow cytometry. (C) Pooled data are presented from (A). (D) The expression of CD62L and CD69 in CD4^+^ T cells, CD8^+^ T cells, NK cells, NKT cells in Figure 2A were analyzed by flow cytometry. (E) Schematic representation of adoptive transfer assay of WT and P2RY12^-/-^ mice. T cells purified by splenic lymphocytes from WT mice and P2RY12^-/-^ mice (8-10 weeks, n = 8) were transferred into Rag1^-/-^ mice through tail vein injection, and 48 hr later, these mice were followed 12.5 mg/kg ConA for 12 hr. (F) The serum levels of AST and ALT were detected. (G) Photomicrographs of representative H&E-stained mouse livers. Massive hepatocyte necrosis (dark arrows) was observed in WT→Rag1^-/-^ mice. (H) The proportion of IFN-γ in CD4^+^ T cells, CD8^+^ T cells from liver lymphocytes of WT→Rag1^-/-^ mice and P2RY12^-/-^→Rag1^-/-^ mice were analyzed by flow cytometry. (I) The serum level of IFN-γ was detected. One representative data of three independent experiments was shown. Data are mean ± SEM. *P< 0.05; **P< 0.01; ***P < 0.001* vs* indicated group. (two-tailed Student's t-test).

The increased level of IFN-γ in mononuclear lymphocytes (including T cells and NK cells) in the liver has been shown to play a key role in ConA-induced immune hepatitis^41^, ^42^. We then determine the effect of P2RY12 knockout on the level of IFN-γ in major subsets of lymphocytes in mice liver with ConA injection, including CD4^+^ T cells (CD3^+^ CD4^+^), CD8^+^ T cells (CD3^+^ CD8^+^), NK cells (CD3^-^ NK1.1^+^), and NKT cells (CD3^+^ NK1.1^+^) (Figure 2A). The number of CD4^+^ T cells and CD8^+^ T cells were found to be significantly reduced in P2RY12^-/-^ mice. In addition, no differences were found in the number of NK cells and NKT cells between WT mice and P2RY12^-/-^ mice (Figure 2B). The proportion of IFN-γ in CD4^+^ T cells, CD8^+^ T cells, and NKT cells in P2RY12^-/-^ mice was lower than those in the WT mice, but there was no marked difference in NK cells (Figure 2C). Our data also indicated that P2RY12 knockout led to T cell expression change of surface markers including downregulation of CD69 and upregulation of CD62L in CD4^+^ T cells and CD8^+^ T cells (Figure 2D). We also investigated the proportion of major subsets of lymphocytes and expression of IFN-γ in spleen and found that there were not dramatic differences in the expression of IFN-γ in the two types of mice (Figure S3A-C).

To further determine whether that the regulatory effect of P2RY12 on IFN-γ expression in T cells was essential for controlling ConA-induced immune hepatitis, the same cellular amount of T cells from P2RY12^-/-^ mice and WT mice were transferred into Rag1^-/-^ mice for 48 hr, followed by ConA injection (Figure 2E, S3D-F). As expected, 12 hr after ConA injection, Rag1^-/-^ mice that received P2RY12^-/-^ T cells showed decreased serum levels of ALT and AST and ameliorated liver damage compared with Rag1^-/-^ mice that received WT T cells (Figure 2F, 2G). The proportions of CD4^+^ IFN-γ^+^ and CD8^+^ IFN-γ^+^ in liver lymphocytes were significantly decreased in Rag1^-/-^ mice that received P2RY12^-/-^ T cells, with reduced serum level of IFN-γ (Figure 2H, I). Additionally, the number of CD4^+^ T cells and CD8^+^ T cells and the proportions of CD4^+^IFN-γ^+^ and CD8^+^IFN-γ^+^ in the spleen were not change of the two groups (Figure S3G-I).

Furthermore, we evaluated the effect of ConA on P2RY12^-/-^ and WT splenocytes* in vitro*. The activity of *in vitro*-generated IFN-γ^+^ cells and expression of cytokines (IFN-γ, IL-6, IL-12p70, TNF-α and IL-10) were also affected by P2RY12 inactivation (Figure S4). Together, these results demonstrate that P2RY12 promotes the activation and effector function of T cells* in vivo* and* in vitro*.

### Ticagrelor and clopidogrel attenuate ConA-induced immune hepatitis

**Figure 3:**
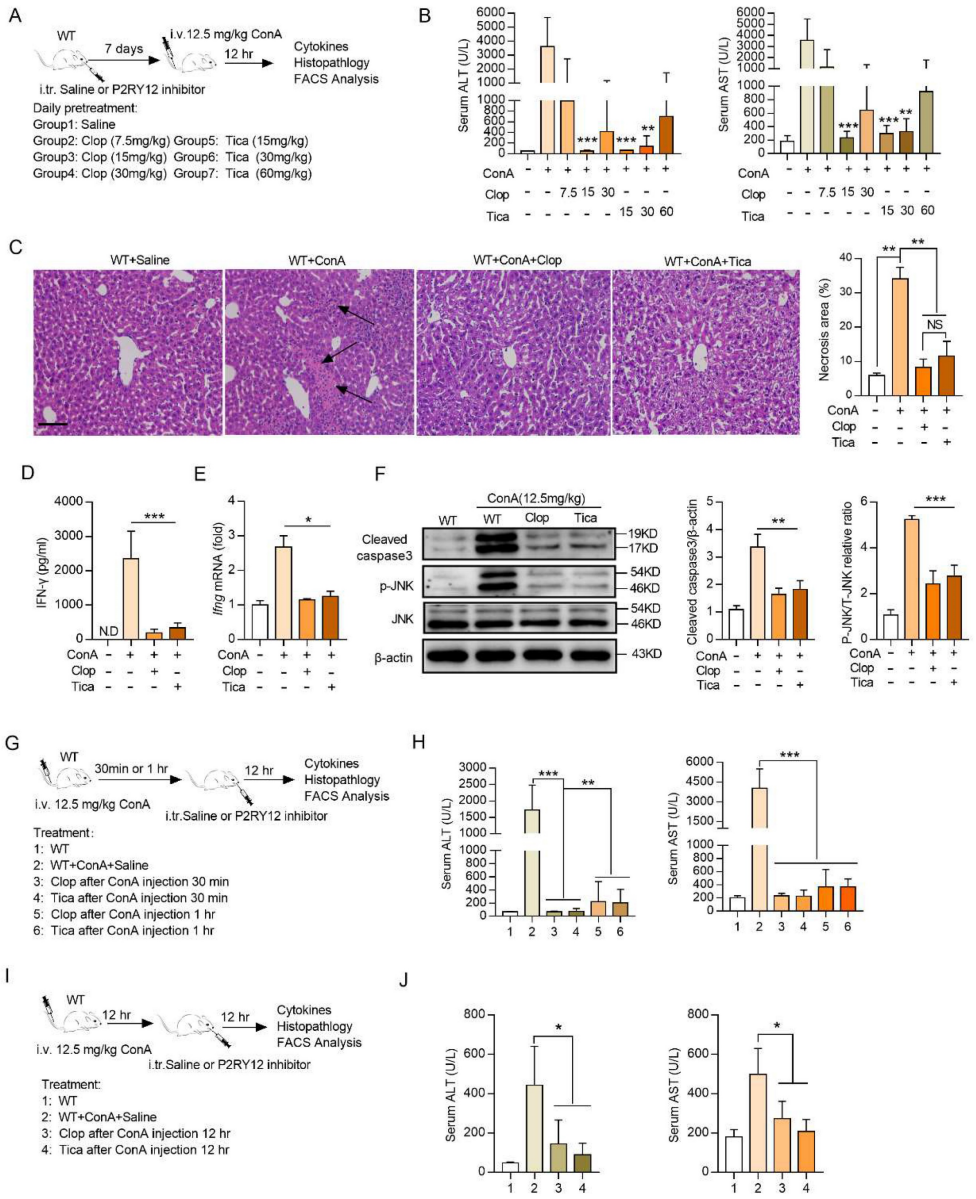
** Clopidogrel and ticagrelor prevent ConA-induced immune hepatitis.** (A) Schematic representation of clopidogrel and ticagrelor prevent ConA-induced immune hepatitis. Different doses of clopidogrel (7.5, 15, 30 mg/kg) or ticagrelor (15, 30, 60 mg/kg) were injected into WT mice via *p.o.* injection (8-10 weeks, n = 5) every day. After 7 days, mice were challenged with ConA (12.5 mg/kg body weight via tail vein injection). (B) Serum levels of ALT and AST were tested simultaneously. (C) Histopathological examination of ConA-induced liver injury. Representative H&E-stained liver sections are shown. Large necrotic areas (dark arrows) were visible in ConA-treated animals and were markedly reduced in mice cotreated with clopidogrel (15 mg/kg) or ticagrelor (15 mg/kg) (left). Pooled data are presented in the right panel. (D) The serum level of IFN-γ and (E) mRNA level of* Ifng* in liver tissue from WT and cotreated with clopidogrel (15 mg/kg) or ticagrelor (15 mg/kg) mice were assessed. (F) The expression levels of cleaved-caspse3 and JNK/p-JNK proteins in ConA-treated WT mice were determined in mouse liver tissues which were injected simultaneously with clopidogrel (15 mg/kg) or ticagrelor (15 mg/kg). (G) Schematic representation of short-term clopidogrel and ticagrelor ameliorate ConA-induced immune hepatitis. Clopidogrel and ticagrelor were given by *p.o.* injection at 15 mg/kg at 30 min or 1 hr after ConA administration, mice were evaluated 12 hr after ConA injection. (H) The serum levels of AST and ALT were detected. (I) Schematic representation of clopidogrel and ticagrelor ameliorates ConA-induced immune hepatitis. Clopidogrel and ticagrelor were given by *p.o.* injection at 15 mg/kg at 12 hr after ConA administration, mice were evaluated 24 hr after ConA injection. (J) The serum levels of AST and ALT were detected. Data are mean ± SEM. *P< 0.05; **P< 0.01; ***P < 0.001 *vs* indicated group. (two-tailed Student's t-test). Data are representative of three independent experiments with similar results.

Ticagrelor and clopidogrel have been shown to selective block the P2RY12 activation^32^. To determine the effects of ticagrelor and clopidogrel on the ConA-induced immune hepatitis* in vivo*, mice were given saline or ticagrelor or clopidogrel for seven consecutive days before ConA injection, respectively (Figure 3A). The results revealed that both ticagrelor (15 mg/kg) and clopidogrel (15 mg/kg) showed the best protective effects in ConA-induced immune hepatitis model, including reducing the serum levels of ALT and AST and alleviating pathological liver injury (Figure 3B, 3C). In addition, ticagrelor or clopidogrel treatment was also showed to reduce the expression of IFN-γ in CD4^+^ T cells, CD8^+^ T cells and NK cells in mice liver (Figure S5), and in serum (Figure 3D). Consistently, ticagrelor or clopidogrel limited *Ifng* mRNA level (Figure 3E), p-JNK and cleaved- caspase3 protein activation in liver tissue (Figure 3F).

We further evaluated whether treatment with ticagrelor or clopidogrel at different time intervals after ConA administration ameliorates ConA-induced immune hepatitis. Ticagrelor or clopidogrel was given by *i.v.* injection at 15 mg/kg at 30 min or 1 hr after ConA injection and mice were evaluated at 12 hr (Figure 3G). Therapeutic administration of ticagrelor or clopidogrel was as effective as the prophylactic treatment, resulting in reducing serum levels of AST and ALT (Figure 3H), as well as liver histological score (Figure S6A). Therapeutic ticagrelor and clopidogrel also inhibited IFN-γ expression in the subsets of CD4^+^ T cells and CD8^+^ T cells of liver lymphocytes (Figure S6B). We further extended the ConA challenge time to 24 hr and administered ticagrelor or clopidogrel at 12 hr after ConA injection (Figure 3I). Treatment with ticagrelor or clopidogrel at 12 hr after ConA injection was as protective as the treatment at 30 min or 1 hr with lower AST and ALT levels (Figure 3J), liver histological score (Figure S6C) and IFN-γ proportion (Figure S6D). Collectively, these results demonstrate that short-term treatment with ticagrelor or clopidogrel can prevent and ameliorate ConA-induced immune hepatitis.

Next, the effects of ticagrelor and clopidogrel in the activation and IFN-γ expression of T cells with ConA treatment were further evaluated *in vitro.* Splenocytes were cultured with different concentrations of ticagrelor and clopidogrel (1, 3, or 10 μM) for 12 hr or 24 hr. The results indicate that ticagrelor and clopidogrel significantly reduce the proportions of CD4^+^IFN-γ^+^ and CD8^+^IFN-γ^+^ (Figure S7).

### P2RY12 alters the metabolomic profile of T cells in liver of mice with ConA-induced immune hepatitis

**Figure 4:**
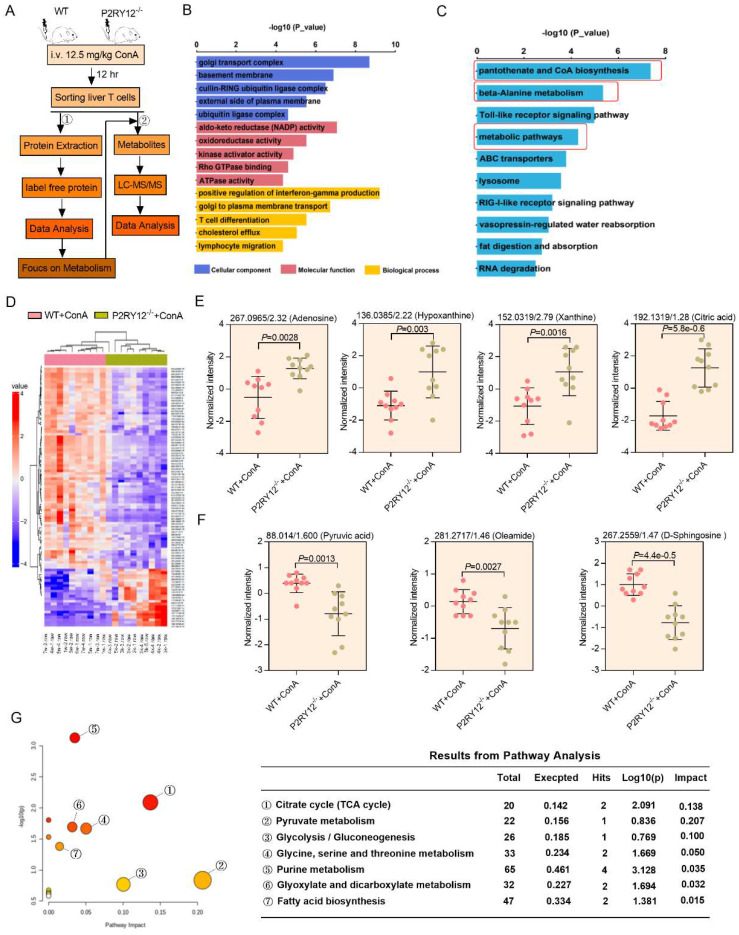
** Multi-omics data of metabolite compositions and metabolic pathways in T cells after ConA administration.** (A) Schematic diagram of the workflow for the analysis of the proteome and metabolome. Hepatic T cells from WT mice and P2RY12^-/-^ mice were induced by ConA. (B) GO enrichment analysis and (C) KEGG enrichment analysis are used to classify the significant differentially expressed proteins in hepatic T cells between WT+ConA mice and P2RY12^-/-^+ConA mice. (D) Heat map analysis of metabolite compositions in hepatic T cells between WT and P2RY12^-/-^ mice induced by ConA. (E, F) Quantitation of the levels of adenosine, hypoxanthine, xanthine, citric acid, oyruvic acid, oleamide, D-sphingosine in hepatic T cells between WT+ConA mice and P2RY12^-/-^+ConA mice. *P*<0.05, fc>1.5 as the significant differentially expressed metabolites. (G) The metabolic pathways identified using Metabo Analyst 3.0 software in hepatic T cells between WT and P2RY12^-/-^ mice induced by ConA. (two-tailed Student's t-test).

To depict the mechanism underlying ConA-induced immune hepatitis, label free-based proteomics analysis on liver T cells from WT mice and P2RY12^-/-^ mice was performed, and 6,294 expressed proteins were identified. For protein differential expression analyses, a false discovery rate (FDR) cut-off of 5% and a 1.5-fold change threshold were used, and 146 proteins were found differentially expressed between two groups (Figure 4A). Gene ontology enrichment analysis revealed that significantly differentially expressed proteins were enriched in the biological processes of “positive regulation of interferon-gamma production”, “cellular carbohydrate metabolic process”, “T cell differentiation” and “lymphocyte migration” (Figure 4B). KEGG pathway enrichment analysis revealed a marked enrichment in cell metabolism pathways (Figure 4C). These findings initially suggest that metabolic reprogramming of T cells may explain the mechanisms by which P2RY12 deficiency protects liver against ConA-induced injury.

We also used untargeted metabolomics to further investigate metabolic mechanisms mediating the effects of P2RY12 in T cells after ConA-injection and identified a total of 30 structurally named metabolite (Figure 4A, 4D). In particular, the levels of adenosine, hypoxanthine, xanthine, while citric acid were significantly increased, and pyruvic acid, oleamide, and D-sphingosine were significantly decreased in the ConA-injected P2RY12^-/-^ group, compared with the WT group (Figure 4E, 4F). Additionally, analysis of the KEGG metabolic library using the Metabo-Analyst (3.0) software revealed that the altered metabolites were enriched in the “tricarboxylic acid cycle (TCA) cycle”, “pyruvate metabolism”, “glycolysis/gluconeogenesis”, “glycine, serine and threonine metabolism”, “purine metabolism”, “glyoxylate and dicarboxylate metabolism”, and “fatty acid biosynthesis” (Figure 4G). Considering the critical role of glucose metabolism in regulating the phenotype and function of T cells, we speculate that P2RY12 may regulate T cell activation and IFN-γ production by driving glucose metabolism reprogramming.

### P2RY12 deletion reduces HK2 expression and aerobic glycolysis in T cells

**Figure 5:**
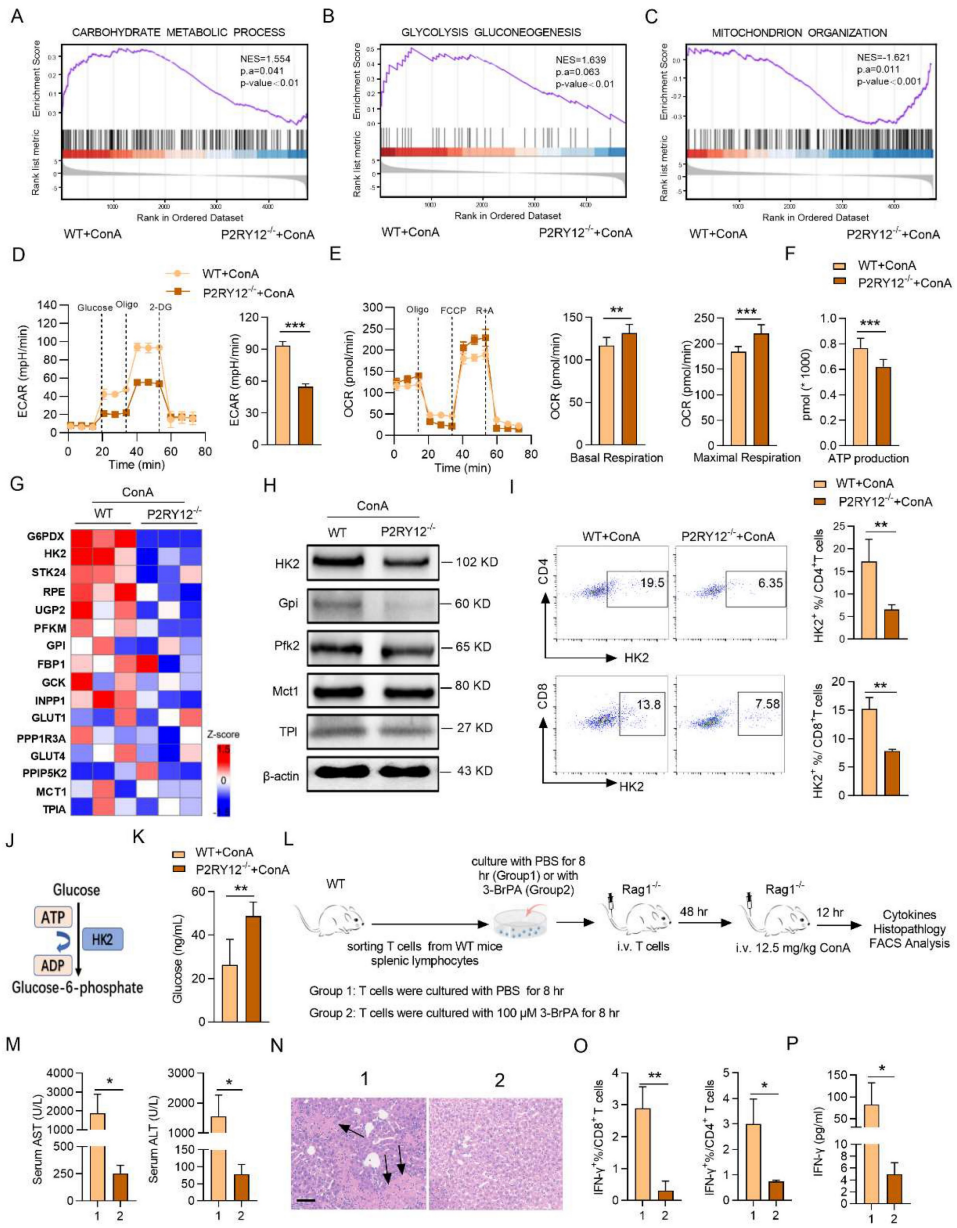
** P2RY12 orchestrates T cells aerobic glycolysis through HK2 in ConA-induced immune hepatitis.** (A-C) GSEA results indicate that genes related to “carbohydrate metabolic process” (A), “glycolysis gluconeogenesis” (B) and “mitochondrion organization” (C) are differentially enriched in hepatic T cells gathered from WT and P2RY12^-/-^ mice after ConA administration 12 hr. NES, normalized enrichment score. FDR, false-discovery rate. (D, E) Seahorse analysis of maximum ECAR (measured after oligomycin injection, Oligo) and baseline OCR and maximum OCR in hepatic T cells from WT mice and P2RY12^-/-^ mice after ConA administration 12 hr. (F) Total intracellular ATP production in hepatic T cells from WT mice and P2RY12^-/-^ mice induced by ConA was measured by ATP-dependent luminescent activity. (G) Heat map depicting changes in the expression of protein involved in “glycolysis” in hepatic T cells. (H) Relative protein expression levels of HK2, GPI, PFK2, MCT1, Tpi and β-actin in hepatic T cells gathered from WT mice and P2RY12^-/-^ mice after ConA administration 12 hr. (I) Flow cytometric analysis of HK2^+^ hepatic T cells gathered from WT mice and P2RY12^-/-^ mice after ConA administration 12 hr. Pooled data are presented in the right panel. (J) Schematic diagram of HK2 function in glycolysis metabolism. (K) Targeted metabolomics analysis the level of glucose in hepatic T cells. (L) Schematic diagram of cell adoptive transfer. T cells from WT mice splenic lymphocytes were co-culture with PBS or 3-BrPA for 8 hr, then transferred into Rag1^-/-^ mice via tail vein injection, followed by challenging these mice with ConA. (M) The serum levels of AST and ALT were detected. (N) Photomicrographs of representative H&E-stained mouse livers. Massive hepatocyte necrosis (dark arrows) was observed in T cells co-culture with PBS mice. (O) The proportion of IFN-γ in CD4^+^ T cells, CD8^+^ T cells from liver lymphocytes. (P) The serum level of IFN-γ was detected. Data are mean ± SEM. *P< 0.05; **P< 0.01; ***P < 0.001 *vs* indicated group. (two-tailed Student's t-test). Data are representative of three independent experiments with similar results.

The gene set enrichment analysis (GSEA) indicated that P2RY12^-/-^ T cells showed reduced enrichment of “glycolysis_gluconeogenesis” and increased enrichment of “mitochondrial organization” (Figure 5A-C). Next, seahorse-based bioenergetics analysis was performed to measure the effect of P2RY12 deletion in the levels of glycolysis and oxidative phosphorylation in T cells. Our data demonstrated that P2RY12 deletion severely reduced the maximum extracellular acidification rate (ECAR), but up-regulated the basal and maximum oxygen consumption rate (OCR) (Figure 5D, 5E). Moreover, the relative ATP production was reduced in ConA-stimulated T cells from the P2RY12^-/-^ mice liver compared with those in the WT mice (Figure 5F). As shown in the heat map, a series of glycolytic enzymes, including G6PDX, HK2, GPI, were down-regulated in CD3^+^ T cells from P2RY12^-/-^ mice based on proteome data (Figure 5G). Western blot analysis further confirmed that the expression of HK2, the first rate-limiting enzyme of glycolysis pathway^43^, ^44^, was reduced in T cell from P2RY12^-/-^ mice liver (Figure 5H). Flow cytometry analysis also showed that CD4^+^ T cells and CD8^+^ T cells from P2RY12^-/-^ mice had a markedly lower level of HK2 expression than those from WT mice (Figure 5I). Consistent with the results of experiments* in vivo*, the HK2 protein level was drastically reduced in CD4^+^ T cells and CD8^+^ T cells treated with ticagrelor or clopidogrel *in vitro* (Figure S8A-D). These findings suggest that glycolysis is positively regulated by P2RY12 and the protein expression of HK2. Since hexokinases (HKs) catalyze the first step of glucose metabolism (Figure 5J), we assessed the contribution of HK2 activity to glucose phosphorylation in liver T cells using targeted metabolomics. Deletion of P2RY12 in liver T cells significantly increased glucose level compared to control group (Figure 5K) but did not change the glucose transport function (Figure S9), indicating that P2RY12 induces HK2 expression and promoted liver T cells glycolysis.

### 3-BrPA represses T cells activation against ConA-induced immune hepatitis

We thus hypothesized that inhibition of HK2 might serve as a feasible therapy to inhibit the expression of IFN-γ and treat ConA-induced immune hepatitis. To test this hypothesis, we examined the effect of the pharmacological inhibition of HK2 with 3-BrPA on ConA-induced immune hepatitis *in vitro* and *in vivo*. The proportions of CD4^+^ IFN-γ^+^ and CD8^+^ IFN-γ^+^ as well as the level of IFN-γ in supernatant were rapidly decreased in splenocytes treated with 3-BrPA for 4 hr (Figure S8E-G). These findings indicate that inhibition HK2 reduces the activation of T cells* in vitro*.

Also, a Rag1^-/-^ transfer model was used to further evaluate the inhibitory effect of 3-BrPA of IFN-γ expression in ConA-induced immune hepatitis* in vivo*. T cells from WT splenocytes were treated with PBS or 3-BrPA (100 μM) and cultured for 8 hr. Then cells were transferred into Rag1^-/-^ mice via tail vein injection, followed by challenging these mice with ConA (Figure 5L). We found that after ConA treatment, the levels of AST and ALT were significantly reduced in 3-BrPA-treated group compared with the control group, with decreased infiltration of inflammatory cells in liver tissue (Figure 5M, 5N). Moreover, we observed a decreased proportion of IFN-γ in CD4^+^ T cells and CD8^+^ T cells, along with a decreased serum level of IFN-γ (Figure 5O, 5P). Together, these results demonstrate that P2RY12 controls the activation and function of CD4^+^ T cells and CD8^+^ T cells via HK2, ultimately leading to the development of immune hepatitis.

### P2RY12 affects HK2 stability through regulating PI3K/Akt signaling pathway and inhibiting lysosomal degradation in T cells

**Figure 6:**
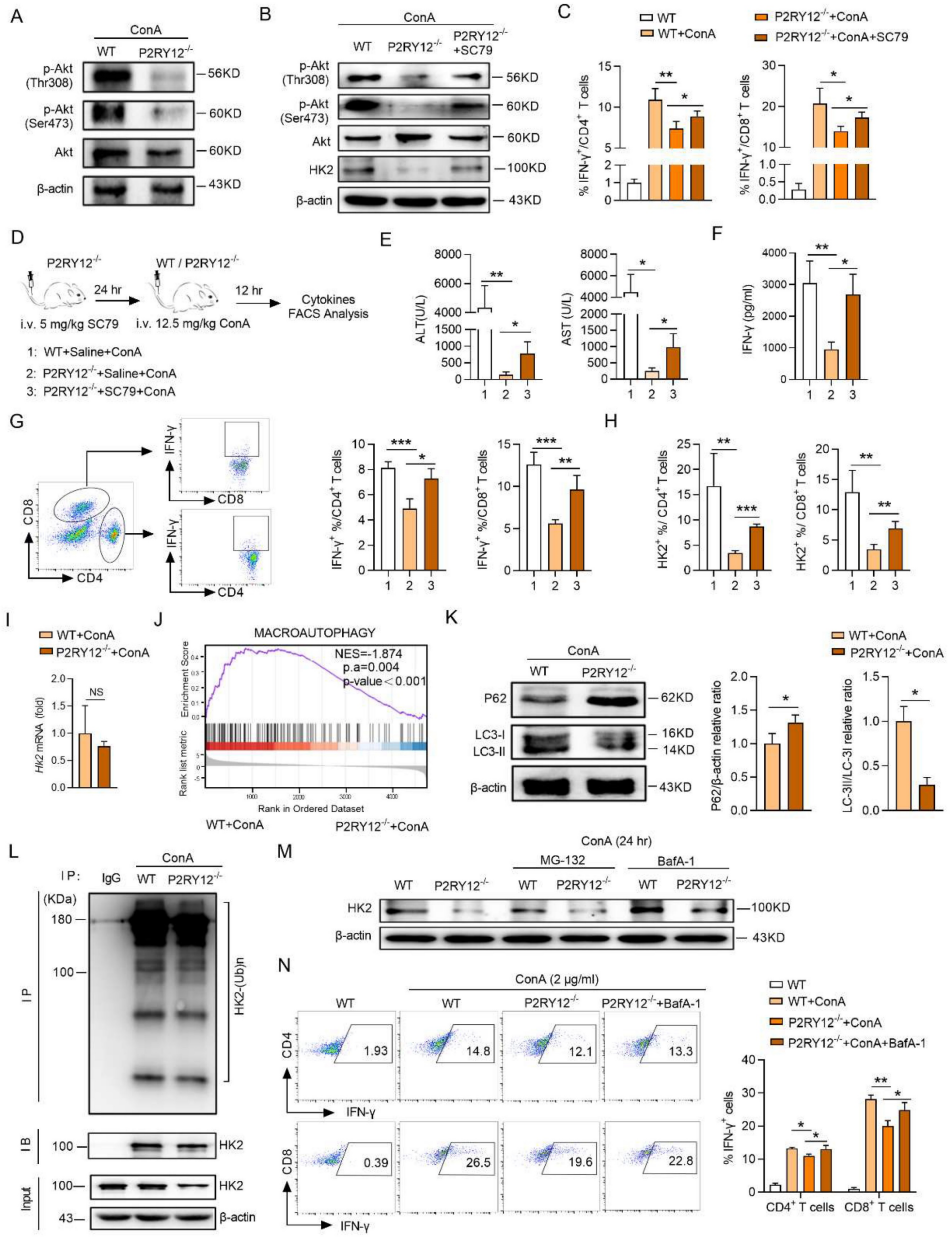
** P2RY12 affects HK2 stability through regulating PI3K/Akt signaling pathway and inhibiting lysosomal degradation in T cells.** (A) Immunoblot analysis of the indicated phosphorylated (p-) and total proteins in WT cells and P2RY12^-/-^ T cells after ConA administration 12 hr. (B) Immunoblot analysis of the indicated HK2, phosphorylated (p-) and total proteins in WT and P2RY12^-/-^ T cells stimulated for 24 hr with ConA followed by incubation with SC79 *in vitro*. (C) Flow cytometric analysis the expression of IFN-γ in T cells from WT T cells and P2RY12^-/-^ T cells activated for 24 hr with ConA followed by incubation with SC79 *in vitro*. (D) Schematic diagram of SC79 aggravates ConA-induced immune hepatitis in P2RY12^-/-^ mice. (E) The serum levels of AST and ALT were detected. (F) The serum level of IFN-γ was detected. The proportion of IFN-γ (G) and HK2 (H) expression in CD4^+^ T cells, CD8^+^ T cells from liver lymphocytes were detected. (I) The mRNA level of *Hk2* was determined in hepatic T cells gathered from WT and P2RY12^-/-^ mice after ConA administration 12 hr. (J) GSEA results indicate that genes related to autophagic pathway enriched in hepatic T cells gathered from WT and P2RY12^-/-^ mice after ConA administration 12 hr. (K) Western blot analysis for the expressions of P62 and LC3 in hepatic T cells. Summary of (K) based on densitometric quantification of three independent experiments (right). (L) Hepatic T cells were gathered from WT and P2RY12^-/-^ mice after ConA administration 12 hr then immunoprecipitated with HK2 antibody. HK2 ubiquitination was determined using anti-ubiquitin antibody. IP: immunoprecipitation, IB: immunoblotting. (M) Western blot analysis of HK2 protein in WT and P2RY12^-/-^ T cells stimulated for 24 hr with ConA followed by incubation with MG-132 and BafA-1* in vitro*. (N) Flow cytometric analysis of IFN-γ in T cells from WT and P2RY12^-/-^ activated for 24 hr with ConA. Pooled data right presented from (N). Data are mean ± SEM. *P< 0.05; **P< 0.01; ***P < 0.001 *vs* indicated group. (two-tailed Student's t-test). Data are representative of three independent experiments with similar results.

The next investigations were focused on revealing the underlying mechanism of P2RY12 deficiency downregulated the protein level of HK2 in T cells. It has been reported that Akt is a critical downstream regulator of P2RY12. Western blot revealed that P2RY12 deletion reduced the activation of Akt indicated by decreasing the phosphorylation-Akt at both Ser473 and Thr308 in ConA-induced immune hepatitis T cells (Figure 6A). Given that SC79 has been shown to specifically enhance the phosphorylation of Akt, we examined the effect of SC79 on HK2 expression in ConA-stimulated P2RY12-deficient T cells *in vitro* and found that treatment with SC79 increased the protein level of HK2 and expression of IFN-γ (Figure 6B, C). This is similar to the result obtained in T cells treated with P2RY12 inhibitors (Figure S10A, S10C). We treated P2RY12^-/-^ mice with SC79 to further examine the role of Akt (Figure 6D). Administrated of SC79 significantly induced serum levels of ALT and AST and the serum level of IFN-γ after ConA-treatment in P2RY12^-/-^ mice (Figure 6E, 6F). Moreover, the expression of IFN-γ and HK2 in hepatic T cells were also significantly upregulated (Figure 6G, 6H).

We further analyzed how PI3K/Akt signaling pathway regulates HK2 stability. PCR analysis indicated that there was no significant difference in the mRNA level of *Hk2* between hepatic T cells from WT mice and P2RY12^-/-^ mice with ConA injection (Figure 6I), Therefore, P2RY12-deficiency did not affect HK2 at the transcriptional level. HK2 acts as a molecular switch from glycolysis to autophagy to ensure cellular energy homeostasis under starvation conditions^45^. Therefore, we investigated whether autophagy would affect protein stability of HK2. Gene enrichment analysis indicated that the pathway of autophagy was inhibited in P2RY12-deficient T cells in the liver of mice injected by ConA (Figure 6J). At the same time, Western blot resulted that P2RY12-deficiency significantly decreased the conversion of LC3-I to LC3-II as well as the increased expression of P62 (Figure 6K).

Since the most prevalent autophagy-targeting signal in mammals is the ubiquitination of cargos, we then measured the ubiquitination level of HK2 by coimmunoprecipitation assay. The results indicated that there was no significant difference in the ubiquitination level of HK2 between the two groups (Figure 6L). Meanwhile, although the defective expression of HK2 in P2RY12-deficient T cells could not be rescued by the proteasome inhibitor MG-132 but it could be partially rescued by the lysosomal inhibitor-BafA1 (Figure 6M). Notable, the proportion of IFN-γ was partly upregulated in P2RY12- deficient T cells treated with lysosomal inhibitor BafA-1 (Figure 6N) (Figure S10B, S10D). These suggest that P2RY12 affects HK2 stability through regulating PI3K/Akt signaling pathway and inhibiting lysosomal degradation in T cells.

### P2RY12 regulates the expression of IFN-γ and HK2 stability in AIH patients and ConA-stimulated healthy human PBMCs *in vitro*

**Figure 7:**
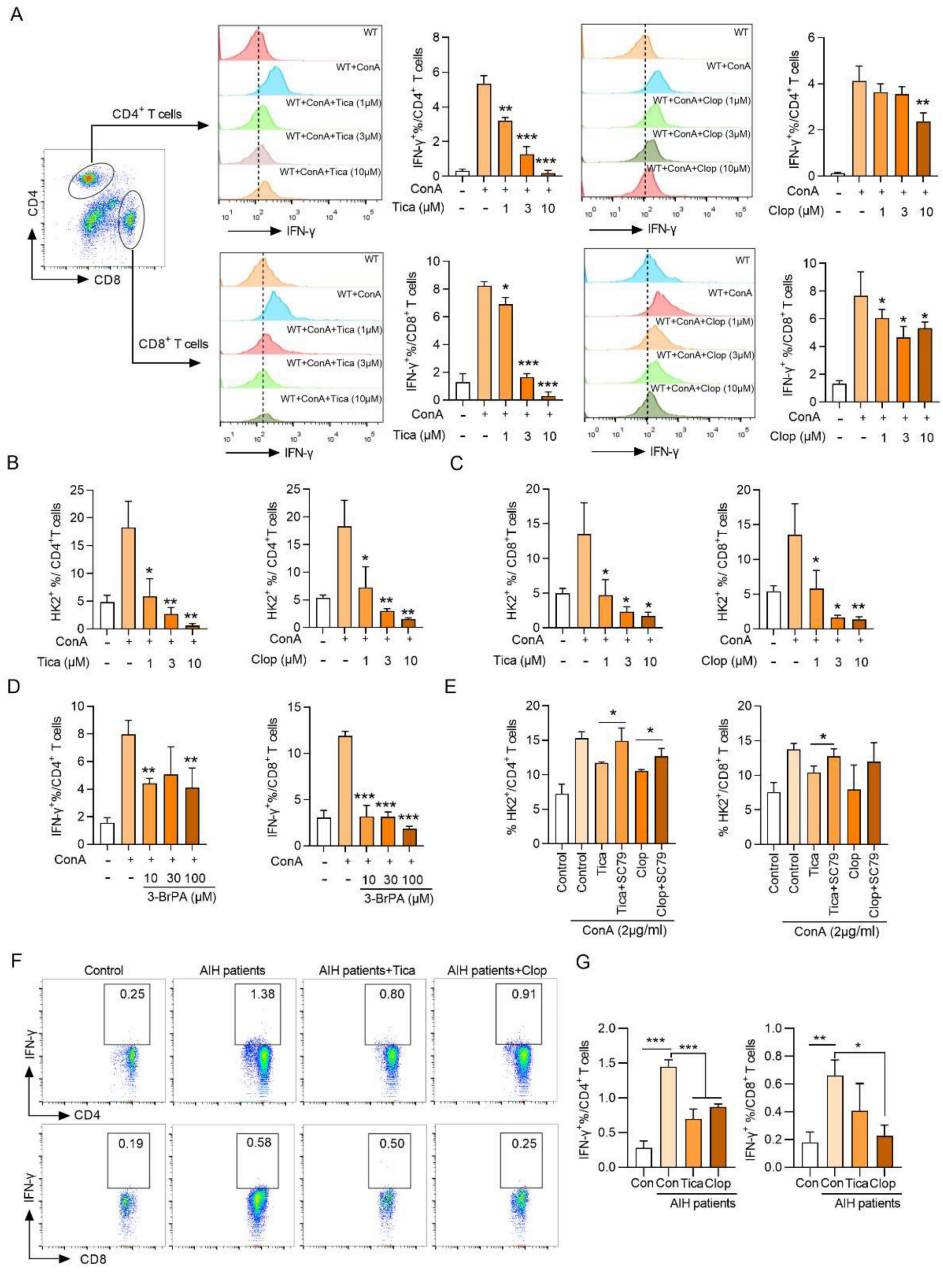
** P2RY12 medicated the expression of IFN-γ and HK2 stability in AIH patients and healthy human PBMCs stimulated by ConA *in vitro*.** (A) Healthy human lymphocytes were obtained and cultured for 24 hr in an environment containing 2 μg/ml ConA and treated with different doses of clopidogrel and ticagrelor (1, 3, 10 μM). Flow cytometric analysis of IFN-γ^+^ in gated CD4^+^ T cells and CD8^+^ T cells. (B, C) Flow cytometric analysis of HK2 in CD4^+^ T cells and CD8^+^ T cells stimulated for 24 hr with ConA followed by incubation with different doses of clopidogrel and ticagrelor. (D) Flow cytometric analysis of IFN-γ^+^ cells in gated CD4^+^ T cells and CD8^+^ T cells in ConA-stimulated human PBMCs treated with 3-BrPA. (E) Flow cytometric analysis of intracellular HK2 in human PBMCs stimulated for 24 hr with ConA followed by incubation with P2RY12 inhibitors (3 μM Clopidogrel or Ticagrelor) or with SC79 for 6 hr. (F) Flow cytometric analysis of IFN-γ^+^ cells in gated CD4^+^ T cells or CD8^+^ T cells in AIH patients PBMCs followed by incubation with P2RY12 inhibitors (3 μM Clopidogrel or Ticagrelor) for 24 hr. (G) Pooled data presented from (F). *P< 0.05; **P< 0.01; ***P < 0.001 *vs* indicated group. Data are representative of three independent experiments. Summary data are shown as mean ± SEM. with P values determined by two-tailed Student's t test.

In order to extend our findings in mice to humans, we investigated whether P2RY12 could regulate the Akt-HK2 axis and IFN-γ production in ConA-stimulated healthy human lymphocytes. The results showed that treatment with different doses of clopidogrel and ticagrelor had a significant dose-dependent inhibitory effect on the levels of IFN-γ and HK2 in CD4^+^ T cells and CD8^+^ T cells (Figure 7A-C).

The measurement of the levels of IFN-γ, IL-6, TNF-α and IL-12p70 in the cell supernatant revealed that clopidogrel and ticagrelor at the dose of 10 μM showed the significant inhibition of the expression of cytokines. Similar findings were also observed at the mRNA levels of four kinds of cytokines (Figure S11). Importantly, the HK2 inhibitor 3-BrPA significantly inhibited the proportion of CD4^+^ IFN-γ^+^ and CD8^+^ IFN-γ^+^ further demonstrated that HK2 is involved in the production of IFN-γ (Figure 7D). In addition, the Akt activator SC79 rescued the decrease of HK2 level caused by P2RY12 inhibition and revealed a crucial role for Akt in regulating the fate of HK2 (Figure 7E).

Moreover, the expression of IFN-γ in AIH patients' PBMCs were also significantly downregulated after the addition of ticagrelor and clopidogrel (Figure 7F, G). These results support the findings that P2RY12 mediates Akt phosphorylation plays an important role in regulating the level of HK2 and, thereby, the function of CD4^+^ T cells and CD8^+^ T cells in AIH patients and healthy human PBMCs *in vitro*.

## Discussion

**Figure 8:**
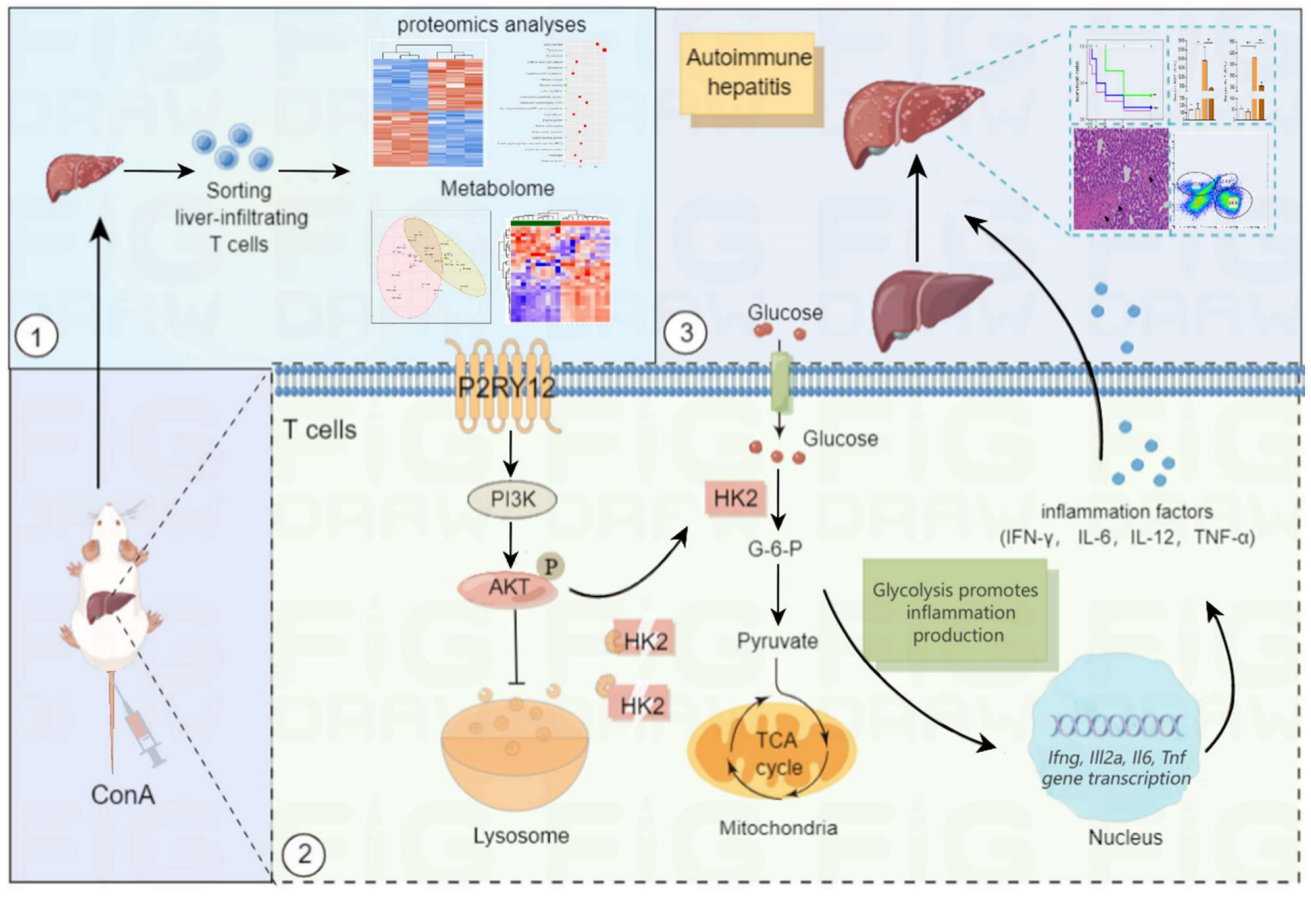
** Summary of the mechanism by purinergic receptor P2Y12 drives ConA-mediated autoimmune hepatitis through hexokinase 2-dependent glycolysis pathway.** Purinergic receptor P2Y12 (P2RY12) affects T cells HK2 stability through regulating PI3K/Akt signaling pathway and inhibiting lysosomal degradation in ConA-mediated autoimmune hepatitis.

In this study, we hypothesized that P2RY12 acts as a novel regulator of T cell metabolism and immune hepatitis. To confirm this hypothesis, the effects of P2RY12 were systematically evaluated *in vitro* and *in vivo*. In addition, combined proteomics and metabolomics analysis were performed to determine the effect of P2RY12 on T cells in ConA-induced immune hepatitis and elucidated the detailed mechanism mediating this effect. We found that P2RY12 triggers glycolysis by promoting glucose uptake in hepatocytes in a ConA-induced immune hepatitis mouse model. P2RY12 functions through a posttranslational mechanism involving the stabilization of HK2 by maintaining the Akt phosphorylation and controlling lysosomal degradation (Figure 8). These findings identify P2RY12 as a pivotal regulator of T cells metabolism and provide a sound theoretical basis for its potential application as a therapeutic target for autoimmune hepatitis.

Previous studies have shown that P2RY12 is involved in multiple biological processes and progression of various diseases 46, 47. Additionally, we have also shown that P2RY12 inhibitor clopidogrel alleviated the clinical symptoms of colitis and diabetes^48^. Our present data suggest that P2RY12 is crucial for T cell responses against ConA-induced immune hepatitis. P2RY12 deficiency mice are considerably less susceptible to ConA-induced immune liver injury. In addition, we found that P2RY12^-/-^ inhibited the activation and function of CD4^+^ T cells and CD8^+^ T cells, rather than those of other immune cells, such as NK cells and NKT cells. Similarly, other researchers have reported that CD4^+^ T cells are a major cellular mediator in the pathology of liver injury^3^, ^49^. Furthermore, the serum levels of pro-inflammation cytokines IFN-γ, IL-6, IL12p70 and TNF-α secreted by immune cells, were decreased in ConA-treated P2RY12^-/-^ mice. Unexpectedly, in ticagrelor and clopidogrel-treated mice, we noticed that P2RY12 inhibitors altered NK cells and NKT cells, indicating that ticagrelor and clopidogrel are connected to the functions of multiple immune cells, different from P2RY12 deficiency.

Glycolysis and mitochondrial OXPHOS have major effects on T cell fate and function^15^, ^50^. Recent studies have proposed that aerobic glycolysis leads to a rapid production of ATP and cytokines in early TCR stimulation^51^. Chang *et al.* also showed that GAPDH binds to the *Ifng* mRNA prior to T cell activation, and TCR-mediated glycolysis dissociates the GAPDH-IFN-γ mRNA complex to promote IFN-γ translation^52^. A major finding of our untargeted metabolomics study is that P2RY12 had a profound effect on intracellular metabolism in T cells. We found that P2RY12 deficiency significantly decreased the level of intracellular glucose and the expression of HK2 in liver-infiltrating T cells. Additional results confirmed that P2RY12 deficiency-induced inhibition of the activation and differentiation of liver infiltrating T cells is mediated by inhibition of the expression of the HK2 protein. Also, using an adoptive cell transfer assay, we found that inhibition of HK2 expression decreased liver-infiltrating T cells involved in IFN-γ production. It has been previously reported that HK2 inhibitor-3-BrPA possessed profound anti-cancer properties by alkylating the active sites of HK2^53^. Additionally, 3-BrPA depleted the intracellular ATP in breast cancer cells whilst induced cytotoxicity and cell apoptosis^54^. In Graft-versus-host disease (GvHD)^55^, 3-BrPA reduced IFN-γ level consistent with our results. Collectively, our findings indicate that 3-BrPA might be a promising candidate for the treatment of diseases associated with T cell activation.

Evidence from previous studies has shown that inhibition of HK2 protects from dextran sodium sulfate (DSS)-induced colitis by decreasing inflammation-induced epithelial cell death^56^. However, other studies suggest a dispensable role for HK2 in mediating T cell responses to acute infections by herpes simplex virus 1 (HSV1) and Armstrong strain of LCMV. Therefore, another major finding of our study is the important role of HK2 in regulating T cell function in ConA-mediated immune hepatitis. A possible explanation is that the discrepancies between our study and previous studies are likely due to the different experimental models used. Our data suggest that P2RY12 deficiency causes HK2 degradation through Akt activation. In this study, we observed that P2RY12 induced Akt phosphorylation, which was indicated by increased the protein levels of phospho-Akt (Ser473 and Thr308). These results are consistent with those reporting that Akt phosphorylation by P2RY12 is observed in vascular smooth muscle cells (VSMCs)^57^. Inactivation of Akt significantly decreased aerobic glycolysis and the protein expression of HK2 in the P2RY12-deficient group. These results suggest that Akt phosphorylation by P2RY12 contributes to aerobic glycolysis by upregulating HK2, thus resulting in the activation and activity of T cells.

In conclusion, our study reveals that P2RY12 is involved in the pathogenesis of ConA-induced immune hepatitis. We also propose that intracellular metabolic reprogramming driven by the HK2/Akt axis is the driving mechanism by which P2RY12 orchestrates ConA-induced immune hepatitis disease. These findings will have implications for the development of future therapeutic approaches involving inhibition of P2RY12 or synergism with therapy target HK2 as a therapeutic tool for the treatment of autoimmune hepatitis.

## Supplementary Material

Supplementary figures and table.

## Abbreviations

ConA: Concanavalin A
HK2: Hexokinase 2
AIH: Autoimmune Hepatitis
DCs: Dendritic Cells
NK: Natural Killer
TNF: Tumor Necrosis Factor
OXPHOS: Oxidative Phosphorylation
FAO: Fatty Acid Oxidation
RA: Rheumatoid Arthritis
SLE: Systemic Lupus Erythematosus
EAE: Experimental Allergic Encephalomyelitis
ALT: Alanine Aminotransferase Test
AST: Aspartate Aminotransferase Test
PBS: Phosphate-buffered Saline
H&E: Hematoxylin and Eosin
PBMCs: Peripheral Blood Mononuclear Cells
RIPA: Radioimmunoprecipitation Assay
SDS-PAGE: Sodium Dodecyl Sulfate-polyacrylamide Gel Electrophoresis
HPLC: High-performance Liquid Chromatography
OCR: Oxygen Consumption Rate
ECAR: Extracellular Acidification Rate
FDR: False Discovery Rate
GvHD: Graft-versus-host Disease
DSS: Dextran Sodium Sulfate
VSMCs: Vascular Smooth Muscle Cells
