# Supplementary material for: Purinergic receptor P2Y12 boosts autoimmune hepatitis through hexokinase 2-dependent glycolysis in T cells
Source: Int J Biol Sci. 2023 Jul 9;19(11):3576–94. doi: 10.7150/ijbs.85133 (PMC10367548; doi:10.7150/ijbs.85133)
Supplement: Supplementary file 1 — Supplementary figures and table. [file ijbsv19p3576s1.pdf]

Supplementary Table 1: Primer sequences used in this study

Gene	Forward (5'-3')	Reverse (5'-3')
m*Il12a*	CCCTGTGCCTTGGTAGCATC	CCTTAGTGTTGATAGCAATGGTGA
m*Il6*	TACCACTTCACAAGTCGGAGGC	CTGCAAGTGCATCATCGTTGTTC
m*Il10*	CGGGAAGACAATAACTGCACCC	CGGTTAGCAGTATGTTGTCCAGC
mT*nf*	GGTGCCTATGTCTCAGCCTCTT	GCCATAGAACTGATGAGAGGGAG
m*Ifng*	CAGCAACAGCAAGGCGAAAAAGG	TTTCCGCTTCCTGAGGCTGGAT
mP2ry12	ATTGACCGCTACCTGAAGAC	GCCTCCTGTTGGTGAGAAT
m*Hk2*	CTAAGGGGTTCAAGTCCAGTGG	AGACCAATCTCGCAGTTCTGA
m*Actb*	CTCCATCCTGGCCTCGCTGT	GCTGTCACCTTCACCGTTCC
h*IFNG*	TCGGTAACTGACTTGAATGTCCA	TCGCTTCCCTGTTTTAGCTGC
h*IL6*	ACTCACCTCTTCAGAACGAATTG	CCATCTTTGGAAGGTTCAGGTTG
h*IL12A*	CCTTGCACTTCTGAAGAGATTGA	ACAGGGCCATCATAAAAGAGGT
h*TNF*	GAGGCCAAGCCCTGGTATG	CGGGCCGATTGATCTCAGC
h*ACTB*	TGAAGTGTGACGTGGACATC	GGAGGAGCAATGATCTTGAT

Supporting Figure

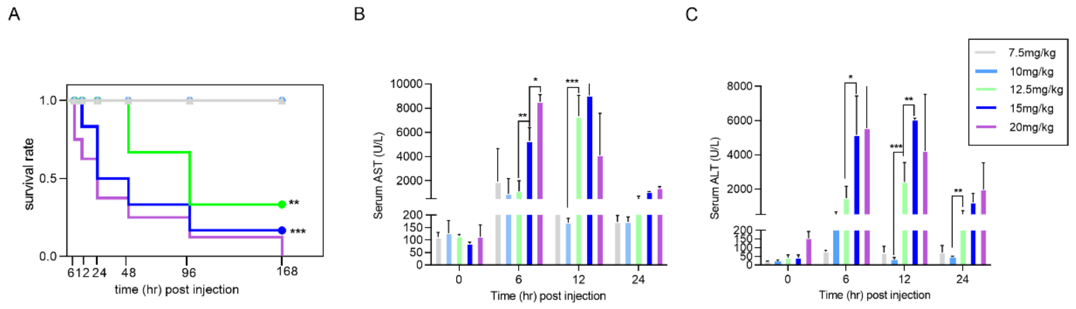

Figure S1. ConA induced experimental autoimmune hepatitis via tail vein injection. WT mice were administered ConA via tail vein injection. (A) Survival rate after different doses of ConA (7.5, 10, 12.5, 15 or 20 mg/kg) injection for 168 hr (n=5). (B) Serum AST level after different doses of ConA (7.5, 10, 12.5, 15 or 20 mg/kg) injection at various time points (0, 6, 12, 24 hr). (C) Serum ALT level after different doses of ConA (7.5, 10, 12.5, 15 or 20 mg/kg) injection at different times (0, 6, 12, 24 hr). One representative data of three independent experiments was shown. Data are mean ± SEM. (two-tailed Student's t-test) *$p < 0.05$; **$p < 0.01$; ***$p < 0.001$.

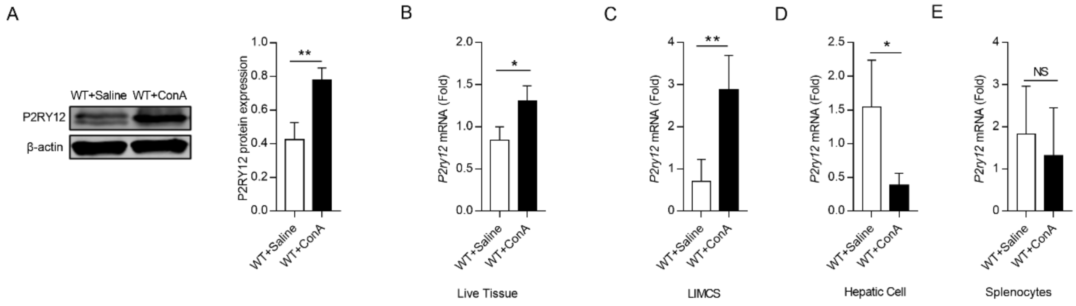

Figure S2. P2RY12 expression in LIMCs and splenic in ConA-induced immune hepatitis. WT mice were administered ConA (12.5 mg/kg body weight via tail vein injection, n=5). 12 hr later, the protein level of P2RY12 in the liver tissue was analyzed by Western Blot (A) and the mRNA levels of *P2ry12* in liver tissue (B), liver tissue-infiltrating mononuclear cells (LIMCs) (C), hepatic cells (D) and splenocytes (E) were analyzed by Real-time PCR assay. One representative data of three independent experiments was shown. Data are mean ± SEM. (two-tailed Student's t-test) *$p < 0.05$; **$p < 0.01$.

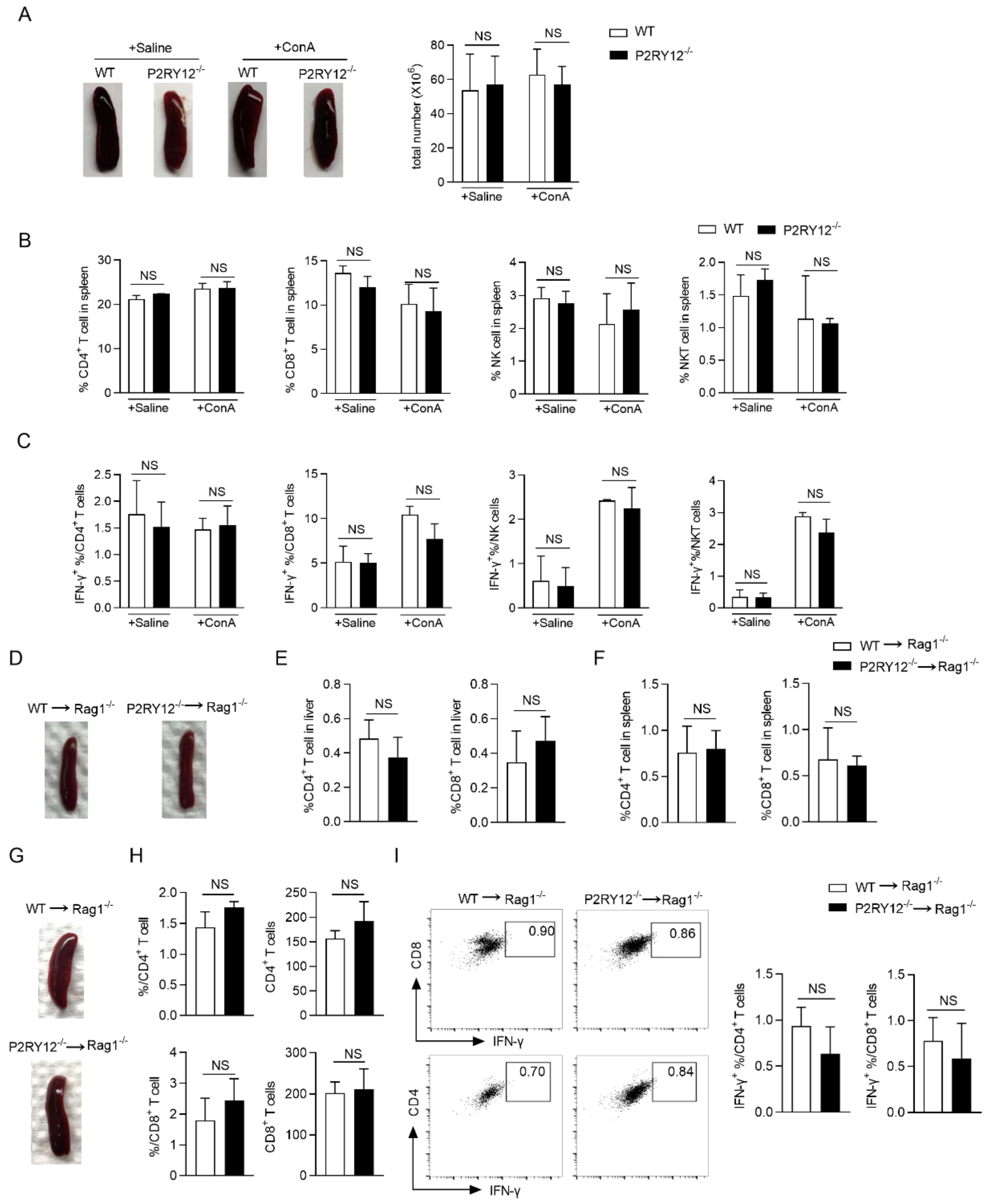

Figure S3. P2RY12 deficiency does not affect the activation and function of splenocytes in ConA-induced immune hepatitis. WT and P2RY12$^{-/-}$ mice were administered with ConA (12.5 mg/kg body weight via tail vein injection, n=5). 12 hr later, the morphology and total number change of spleen were observed (A). (B) The percentage of CD4^{+} T cells, CD8^{+} T cells, NK cells, NKT cells in splenocytes was detected by FCM and analyzed. (C) The proportion of IFN-γ in CD4^{+} T cells, CD8^{+} T cells, NK cells, NKT cells from spleen were analyzed by FCM and calculated. (D) The morphology of spleen before ConA injection was observed. (E) The percentage and total number of CD4^{+} T cells and CD8^{+} T cells in liver before ConA injection was detected by FCM. (F) The percentage and total number

of CD4$^+$ T cells and CD8$^+$ T cells in splenocytes before ConA injection was detected by FCM. (G) The morphology of spleen after ConA injection was observed. (H) The percentage and total number of CD4$^+$ T cells and CD8$^+$ T cells in splenocytes after ConA injection was detected by FCM. (I) Flow cytometric analysis of IFN-γ$^+$ in splenocytes after ConA injection. Pooled data are presented in the right panel. One representative data of three independent experiments was shown. Data are mean ± SEM. (two-tailed Student's t-test)

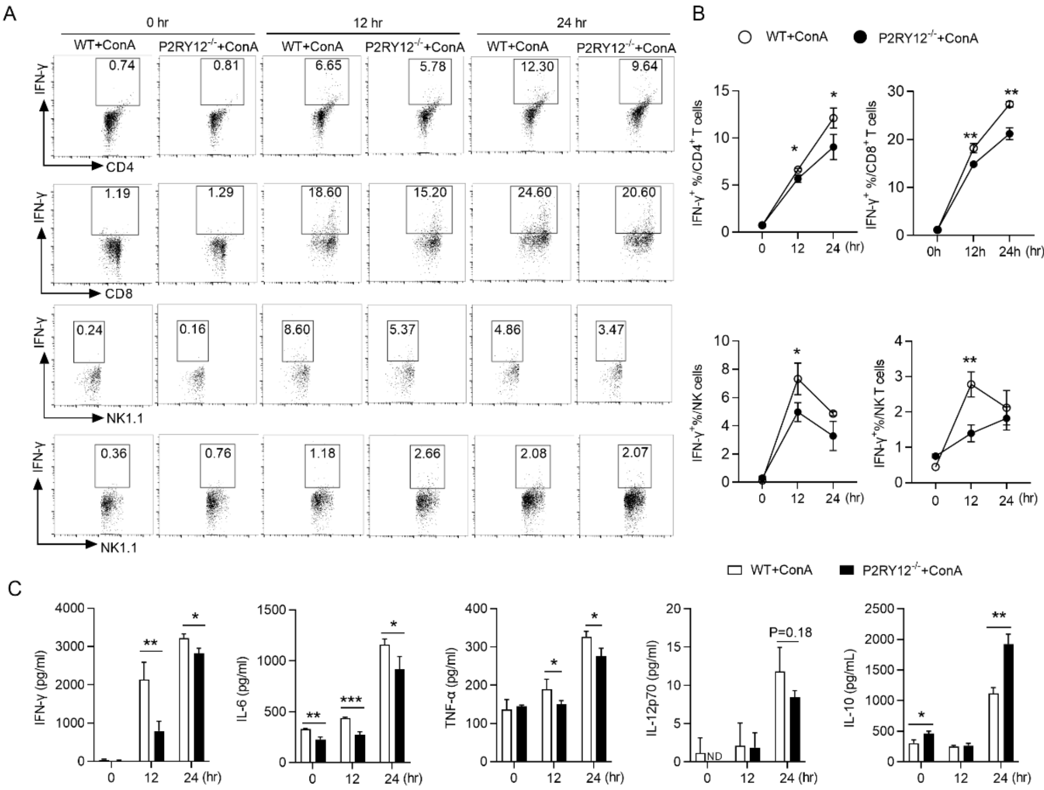

Figure S4. P2RY12 deficiency reduces the proportion of IFN-γ$^+$ and the production of inflammatory cytokines *in vitro*. (A) Splenocytes obtained from 7-week-old WT and P2RY12$^{-/-}$ mice were cultured for 0, 12 and 24 hr in an environment containing 2 μg/ml ConA. (A) Representative FACS images showing the IFN-γ expression in CD4$^+$ T cells, CD8$^+$ T cells, NK cells, and NKT cells. (B) Statistical data for IFN-γ percentage of CD4$^+$ T cells, CD8$^+$ T cells, NK cells, and NKT cells. (C) ELISA assays IFN-γ, IL-6, TNF-α, IL-12p70 and IL-10 levels in cell supernatants. Data are mean ± SEM. (two-tailed Student's t-test) *$p < 0.05$; **$p < 0.01$.

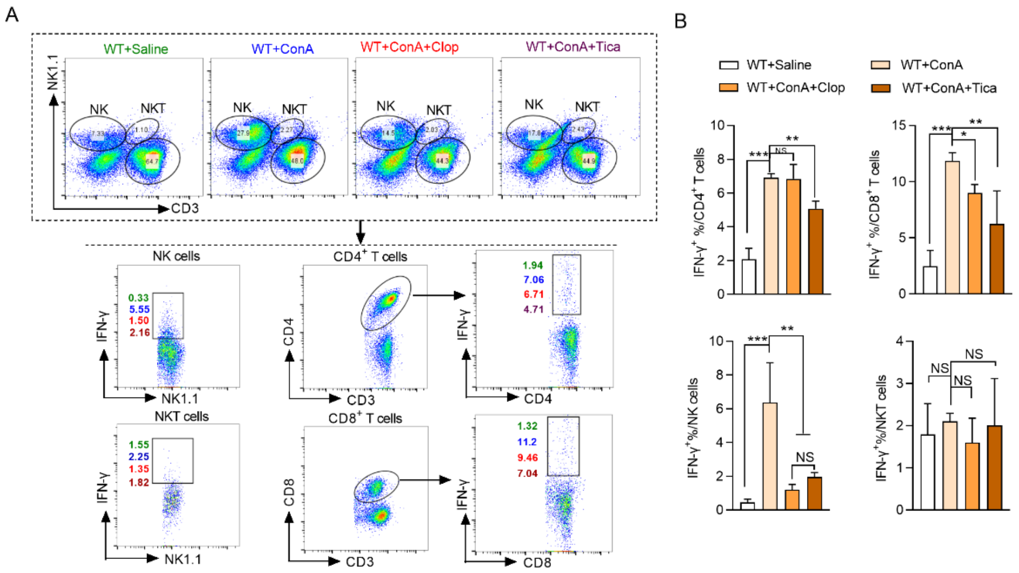

Figure S5. Clopidogrel and Ticagrelor attenuate the percentage of IFN-γ$^+$ cells *in vivo*. Clopidogrel (15 mg/kg) or Ticagrelor (15 mg/kg) were injected into WT mice via p.o. injection (8-10 weeks, n = 5) every day. After 7 days, mice were challenged ConA (12.5 mg/kg body weight via tail vein injection). (A) Flow cytometry analyzes IFN-γ expression in CD4$^+$ T cells, CD8$^+$ T cells, NK cells, and NKT cells from liver lymphocytes. (B) Statistical data for IFN-γ percentage of CD4$^+$ T cells, CD8$^+$ T cells, NK cells, and NKT cells. One representative data of three independent experiments was shown. Data are mean ± SEM. (two-tailed Student's t-test) *$p < 0.05$; **$p < 0.01$; ***$p < 0.001$.

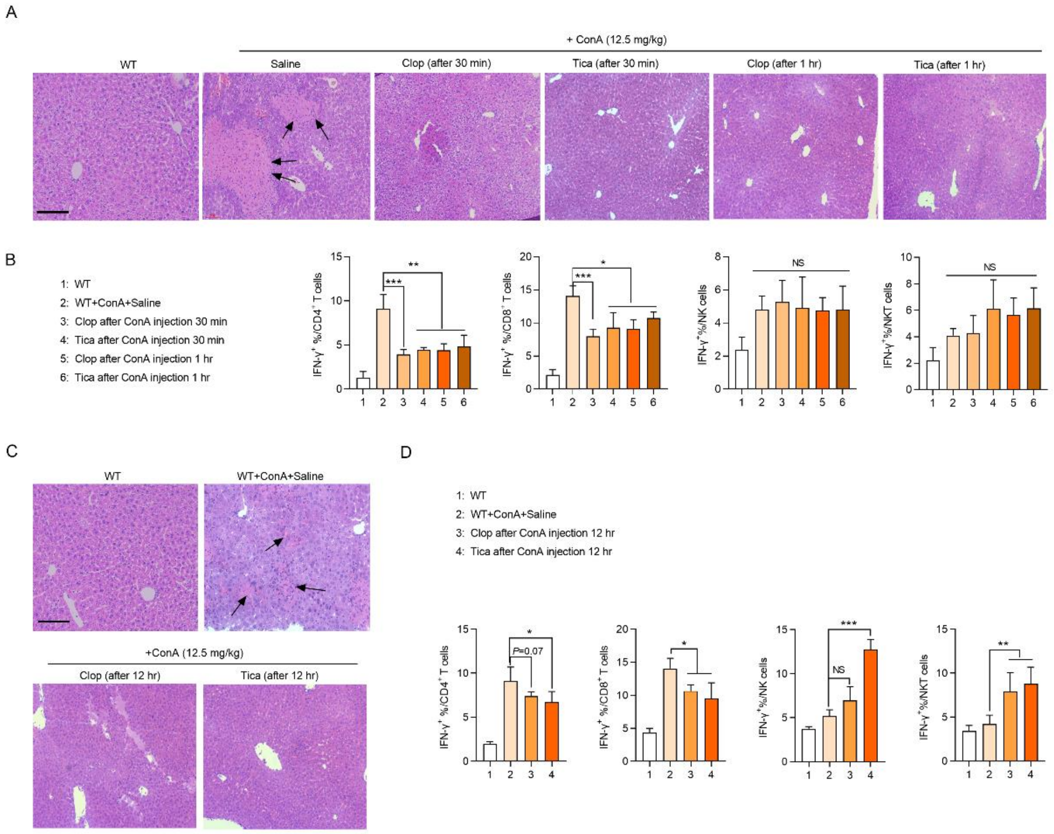

Figure S6. Clopidogrel and ticagrelor alleviate ConA-induced immune hepatitis. Clopidogrel and ticagrelor were given by *p.o.* injection at 15 mg/kg at 30 min or 1 hr after ConA administration, mice were evaluated 12 hr or 24 hr after ConA injection. (A) Histopathological examination of ConA-induced liver injury. Representative H&E-stained liver sections are shown. Large necrotic areas (dark arrows) were visible in ConA-treated animals and were markedly reduced in mice. (B) The proportion of IFN-γ^{+} in $CD4^{+}$ T cells, $CD8^{+}$ T cells, NK cells, NKT cells were examined by using intracellular staining. (C) Clopidogrel and ticagrelor ameliorates ConA-induced immune hepatitis. Clopidogrel and ticagrelor were given by *p.o.* injection at 15 mg/kg at 12 hr after ConA administration, mice were evaluated 24 hr after ConA injection. Representative H&E-stained liver sections are shown. Large necrotic areas (dark arrows) were visible in ConA-treated animals and were markedly reduced in mice. (D) The proportion of IFN-γ^{+} in $CD4^{+}$ T cells, $CD8^{+}$ T cells, NK cells, NKT cells were examined by using intracellular staining. One representative data of three independent experiments was shown. Data are mean ± SEM. (two-tailed Student's t-test) *$p < 0.05$; **$p < 0.01$; ***$p < 0.001$.

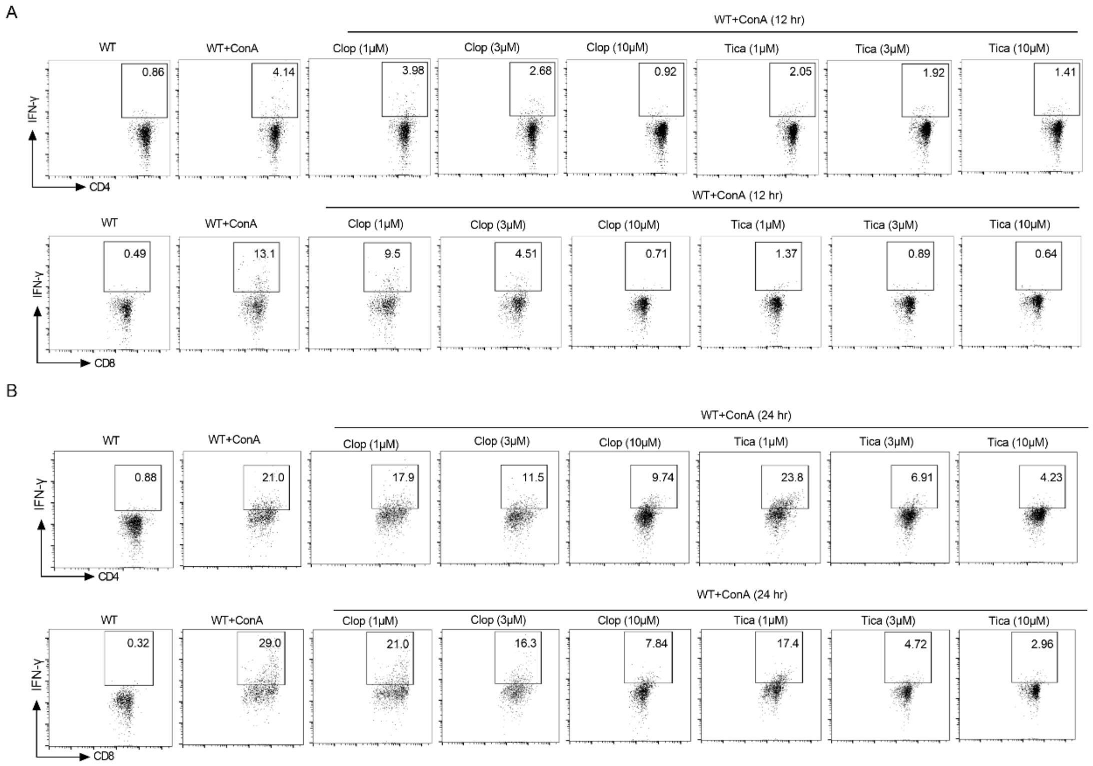

Figure S7. Clopidogrel and ticagrelor reduces the proportion of IFN-γ⁺ T cells *in vitro*. Splenocytes obtained from 7-week-old C57BL/6 mice were cultured for 12 hr or 24 hr in an environment containing 2 μg/ml ConA and treated with different doses of clopidogrel and ticagrelor (1, 3 or 10 μM), except for the control group. Representative FACS images showing percentage of IFN-γ^+ in $CD4^+$ T cells or $CD8^+$ T cells in 12 hr (A) and 24 hr (B).

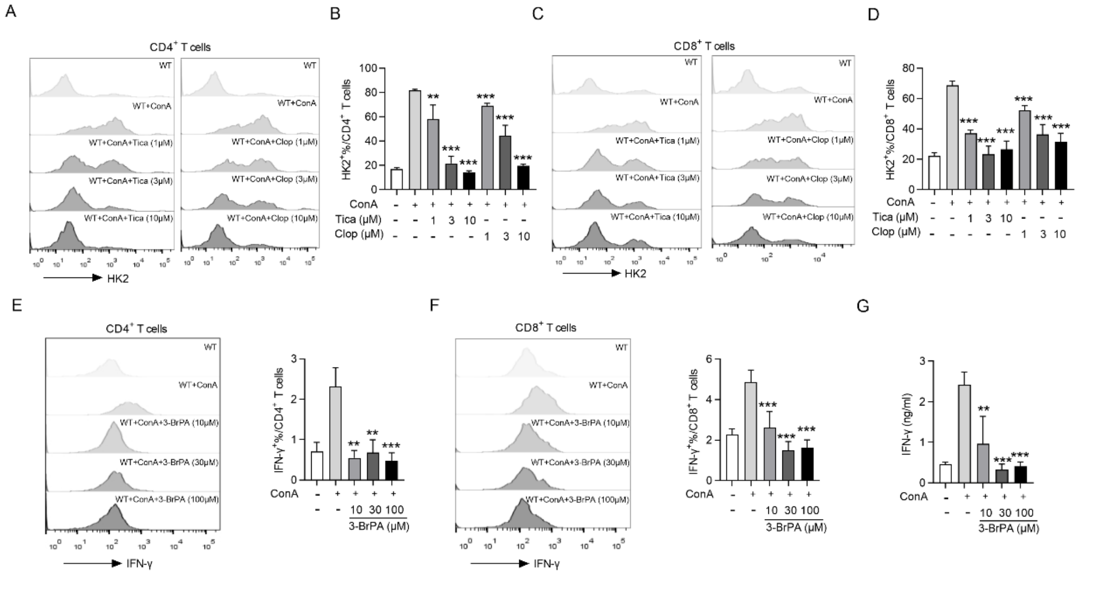

Figure S8. HK2 is required for ConA-induced IFN-γ expression. Splenocytes obtained from

7-week-old C57BL/6 mice were cultured for 24 hr in an environment containing 2 μg/ml ConA and treated with different doses of clopidogrel and ticagrelor (1, 3, 10 μM), except for the control group. (A, C) Flow cytometry analyzes HK2 expression in $CD4^+$ T cells and $CD8^+$ T cells. (B, D) Statistical data for HK2 percentage of $CD4^+$ T cells and $CD8^+$ T cells. Splenocytes obtained from 7-week-old C57BL/6 mice were cultured for 24 hr in an environment containing 2 μg/ml ConA and treated with different doses of 3-BrPA (10, 30, 100 μM) except for the control group. (E, F) Flow cytometry analyzes IFN-γ expression in $CD4^+$ T cells and $CD8^+$ T cells. (G) ELISA assays IFN-γ level in cell supernatants. Data are mean ± SEM. (two-tailed Student's t-test) *$p < 0.05$; **$p < 0.01$; ***$p < 0.001$.

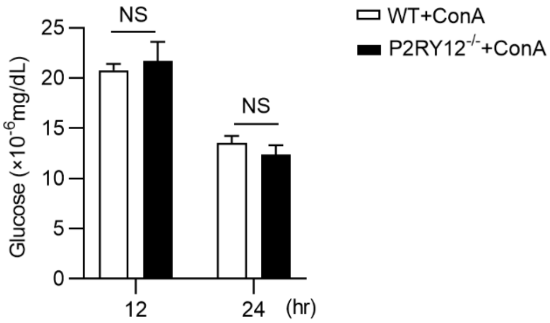

Figure S9. P2RY12 does not change glucose transport into T cells. Splenocytes obtained from 7-week-old WT and P2RY12$^{-/-}$ mice were cultured for 24 hr in an environment containing 2 μg/ml ConA. Glucose level in cell supernatants was measured by glucose-dependent luminescent activity. Data are mean ± SEM. (two-tailed Student's t-test). $^{NS}p > 0.05$.

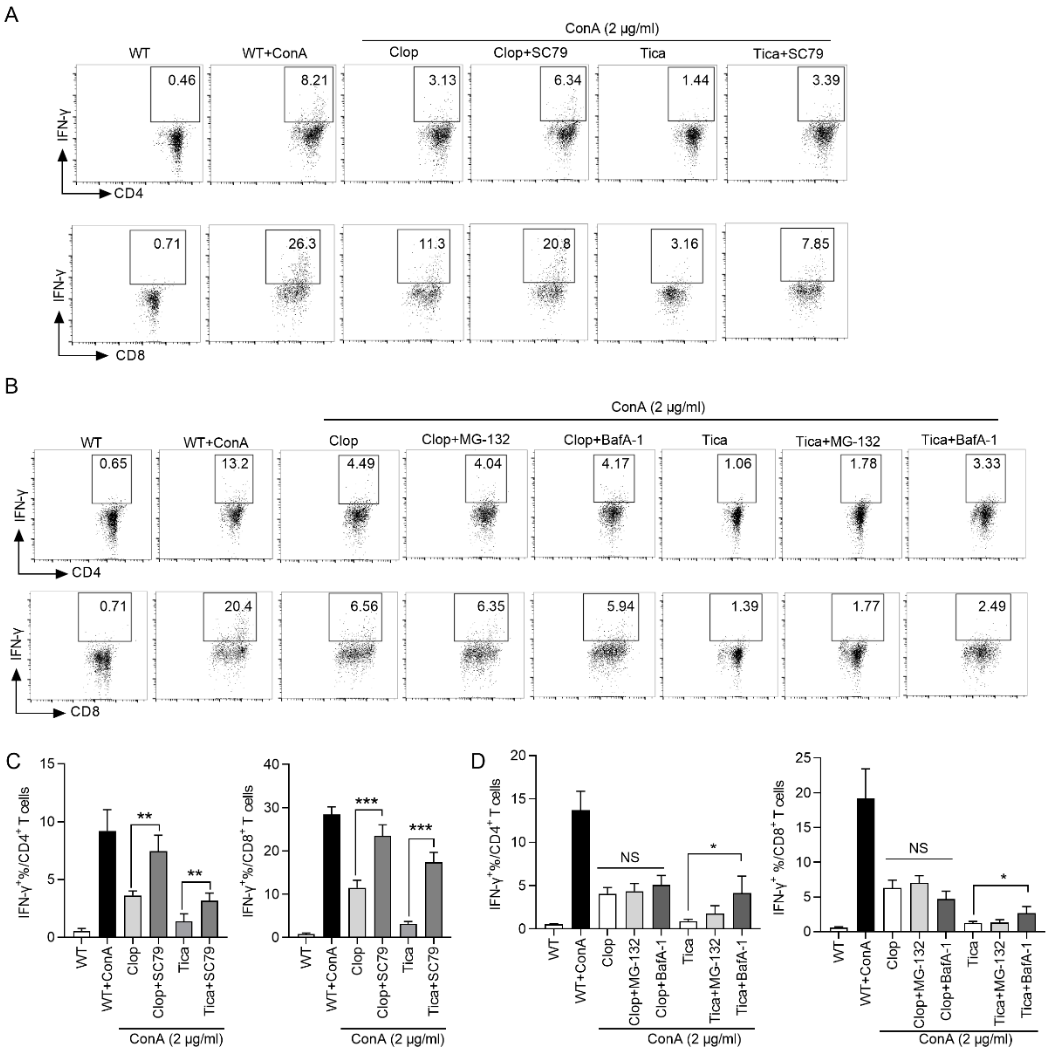

Figure S10. Clopidogrel and ticagrelor regulate the expression of IFN-γ through inhibiting Akt phosphorylation and activating lysosomal degradation. (A, C) Splenocytes obtained from 7-week-old C57BL/6 mice were cultured for 24 hr in an environment containing 2 μg/ml ConA and treated with 3 μM clopidogrel or ticagrelor with administered SC-79. The proportion of IFN-γ^+ in CD4$^+$ T cells and CD8$^+$ T cells were examined by using intracellular flow cytometry staining. (B, D) Splenocytes obtained from 7-week-old C57BL/6 mice were cultured for 24 hr in an environment containing 2 μg/ml ConA and treated with 3 μM clopidogrel or ticagrelor with administered MG-132 or BafA-1, except for the control group. The proportion of IFN-γ^+ in CD4$^+$ T cells, CD8$^+$ T cells were examined by using intracellular flow cytometry staining. Data are mean ± SEM. (two-tailed Student's t-test) *$p < 0.05$; **$p < 0.01$; ***$p < 0.001$.

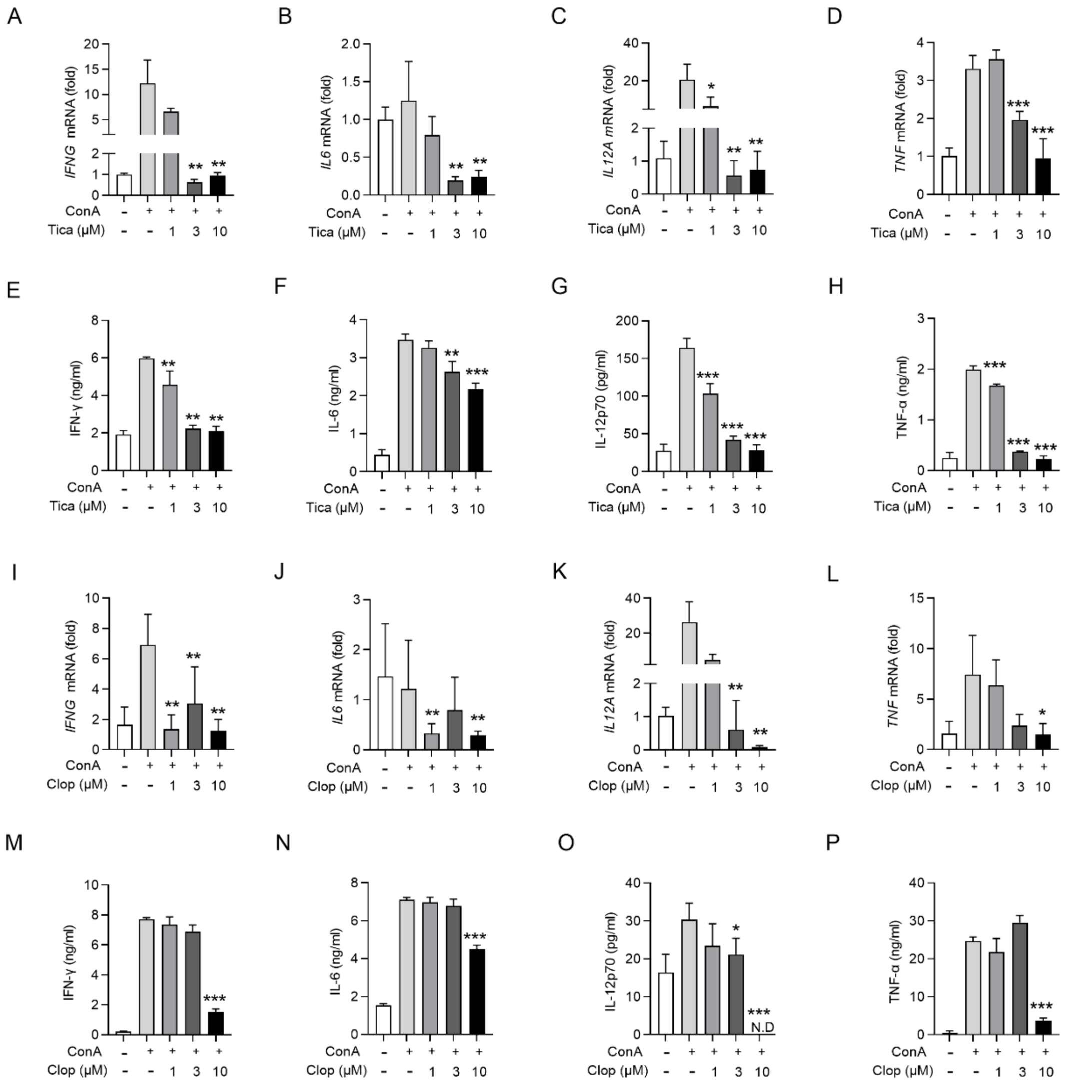

Figure S11. Clopidogrel and ticagrelor ameliorate production of inflammatory cytokines in healthy human PBMCs stimulated by ConA *in vitro*. Healthy human PBMCs of healthy individual and cultured for 24 hr in an environment containing 2 μg/ml ConA and treated with different doses of Clopidogrel and Ticagrelor (1, 3, 10 μM), except for the control group. The mRNA expression levels of (A) *IFNG*, (B) *IL6*, (C) *IL12A* and (D) *TNF* in ConA-stimulated PBMCs treated with Ticagrelor or nothing for 24 hr. Statistical analysis of (E) IFN-γ, (F) IL-6, (G) IL-12p70 and (H) TNF-α levels in cell supernatants. The mRNA expression levels of (I) *IFNG*, (J) *IL6*, (K) *IL12A* and (L) *TNF* in ConA-stimulated PBMCs treated with Clopidogrel or nothing for 24 hr. Statistical analysis of (M) IFN-γ, (N) IL-6, (O) IL-12p70 and (P) TNF-α levels in cell supernatants. Data are mean ± SEM. (two-tailed Student's t-test) *$p < 0.05$; **$p < 0.01$; ***$p < 0.001$.
